# Learning with Slight Forgetting Optimizes Sensorimotor Transformation in Redundant Motor Systems

**DOI:** 10.1371/journal.pcbi.1002590

**Published:** 2012-06-28

**Authors:** Masaya Hirashima, Daichi Nozaki

**Affiliations:** Division of Physical and Health Education, Graduate School of Education, The University of Tokyo, Bunkyo-ku, Tokyo, Japan; University College London, United Kingdom

## Abstract

Recent theoretical studies have proposed that the redundant motor system in humans achieves well-organized stereotypical movements by minimizing motor effort cost and motor error. However, it is unclear how this optimization process is implemented in the brain, presumably because conventional schemes have assumed a priori that the brain somehow constructs the optimal motor command, and largely ignored the underlying trial-by-trial learning process. In contrast, recent studies focusing on the trial-by-trial modification of motor commands based on error information suggested that forgetting (i.e., memory decay), which is usually considered as an inconvenient factor in motor learning, plays an important role in minimizing the motor effort cost. Here, we examine whether trial-by-trial error-feedback learning with slight forgetting could minimize the motor effort and error in a highly redundant neural network for sensorimotor transformation and whether it could predict the stereotypical activation patterns observed in primary motor cortex (M1) neurons. First, using a simple linear neural network model, we theoretically demonstrated that: 1) this algorithm consistently leads the neural network to converge at a unique optimal state; 2) the biomechanical properties of the musculoskeletal system necessarily determine the distribution of the preferred directions (PD; the direction in which the neuron is maximally active) of M1 neurons; and 3) the bias of the PDs is steadily formed during the minimization of the motor effort. Furthermore, using a non-linear network model with realistic musculoskeletal data, we demonstrated numerically that this algorithm could consistently reproduce the PD distribution observed in various motor tasks, including two-dimensional isometric torque production, two-dimensional reaching, and even three-dimensional reaching tasks. These results may suggest that slight forgetting in the sensorimotor transformation network is responsible for solving the redundancy problem in motor control.

## Author Summary

It is thought that the brain can optimize motor commands to produce efficient movements; however, it is unknown how this optimization process is implemented in the brain. Here we examine a biologically plausible hypothesis in which slight forgetting in the motor learning process plays an important role in the optimization process. Using a neural network model for motor learning, we initially theoretically demonstrated that motor learning with a slight forgetting factor consistently led the network to converge at an optimal state. In addition, by applying the forgetting scheme to a more sophisticated neural network model with realistic musculoskeletal data, we showed that the model could account for the reported stereotypical activity patterns of muscles and motor cortex neurons in various motor tasks. Our results support the hypothesis that slight forgetting, which is conventionally considered to diminish motor learning performance, plays a crucial role in the optimization process of the redundant motor system.

## Introduction

The motor system exhibits tremendous redundancy [1]. For example, an infinite number of muscle activation patterns can generate a desired joint torque because multiple muscles span a single joint; moreover, several combinations of neuronal activity in the motor cortex can achieve exactly the same muscle activation pattern. Nevertheless, strongly stereotypical patterns are observed in the activity patterns of neurons in the primary motor cortex (M1) [2]–[6] as well as those of the muscles [7]–[11]. How, then, does the motor system select such stereotypical behavior from an infinite number of possible solutions?

The hypothesis that the brain selects a solution that minimizes the cost of movement has long been proposed [12], [13]. Recent studies have indicated that various aspects of motor control, such as trajectory formation and the selection of a muscle activation pattern, can be reproduced when the motor command is constructed to minimize the cost *J* [10], [14], [15], as expressed by:
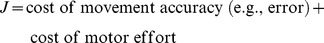
(1)With regard to the movement accuracy, it is widely accepted that information on movement error is available to the brain [16]–[21]. In contrast, there is no evidence indicating that the brain explicitly computes the cost of motor effort across a vast number of neurons and muscles (i.e., the sum of the squared activity) [22]. Some theoretical studies have proposed that the brain can implicitly minimize the motor effort cost by minimizing the variance of motor performance in the presence of signal-dependent noise (SDN) [23], [24]. This theory has attracted widespread interest because the minimization of variance is more biologically plausible than the explicit minimization of the motor effort cost. However, there is still no evidence indicating that a statistical quantity such as variance is represented in the brain [25], [26]. Thus, it is unknown how the optimization process that minimizes the cost function *J* is implemented in the brain.

It should be noted that these conventional optimization studies tacitly assume that the brain somehow constructs a motor command that theoretically minimizes the cost function, and largely ignored the underlying trial-by-trial learning process [8]–[13], . In contrast, recent studies that focused on the trial-by-trial modification of motor commands suggested that forgetting (i.e., synaptic weight decay) is helpful for minimizing the motor effort cost without an explicit calculation of a complex quantity (i.e., sum of squares) [29]–[35]. Although the “weight decay method” has been used as a technical method in the machine-learning community since the 1980s to suppress irrelevant connections in a neural network and to improve the network's generalization ability [36]–[39], it is only recently that its potential for solving the redundancy problem in the context of motor control began to be investigated. Importantly, Emken et al. [32] demonstrated that trial-by-trial error-feedback learning with forgetting minimizes a cost function that is the weighted sum of motor error and motor effort. However, since the authors formulated their motor learning scheme with only a single lumped muscle (i.e., a non-redundant actuator), their model cannot predict the activation patterns of individual muscles. Burdet et al. and Franklin et al. also proposed a similar but more elaborate algorithm (the V-shaped learning function) and showed that it could predict the evolution of the activity of individual muscles that was actually observed when human subjects learn to perform movements in novel dynamic environments [29], [34], [35]. This algorithm has been also used to realize human-like adaptive behavior in robots [40], [41].

However, it is unknown whether the decay algorithm could minimize the cost (*J*) in a highly redundant neural network that includes M1 neurons and whether it can predict the activation patterns of M1 neurons. Neurophysiological studies reported that the preferred direction (PD; the direction in which the neuron is maximally active) of M1 neurons was stereotypically biased toward a specific direction [2]–[6]. Although a conventional optimization study suggested that the bias is a result of the minimization of the cost (*J*) [27], it is unclear how the two terms of the cost function (i.e., error and effort) are minimized on a trial-by-trial basis and how the PD bias of M1 neurons is formed during the optimization process.

To gain insight into these mechanisms, we conducted computer simulations of motor learning by applying the “feedback-with-decay” algorithm to a redundant neural network model for sensorimotor transformation. First, we used a simple linear model to gain a firm theoretical understanding of the effect of the decay on the minimization of the cost (*J*) and the formation of the PD bias. Then, using a non-linear network model with realistic musculoskeletal data, we examined numerically whether this algorithm could predict the PD bias reported in various motor tasks. Our simulations revealed that the “feedback-with-decay” algorithm could consistently reproduce the PD distribution observed during various motor tasks, including a 2D isometric torque production task and a reaching task, and even a 3D reaching task.

## Results

### Linear neural network model

**Figure 1:**
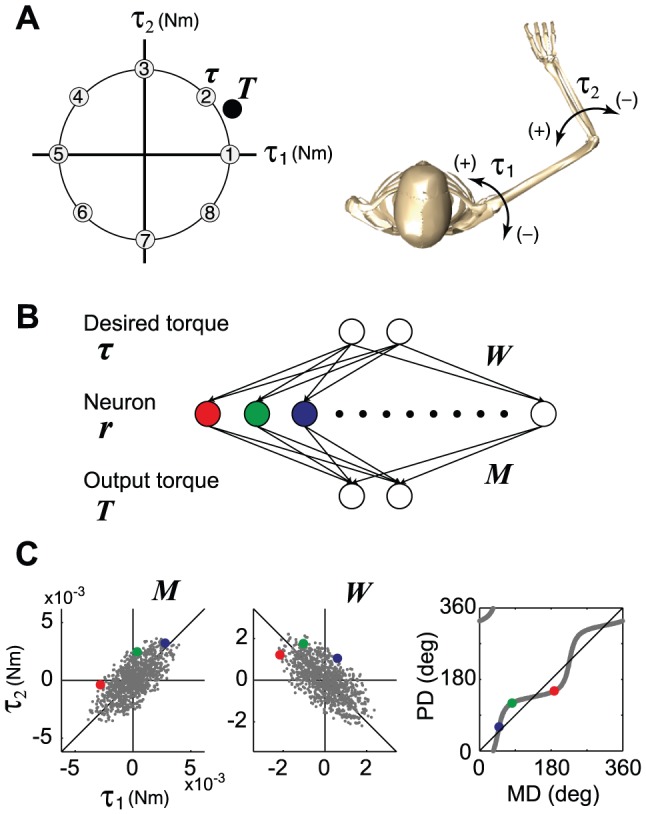
Motor task and redundant neural network. (**A**) Eight targets that were uniformly distributed in the 2D torque plane were used to train the network. (**B**) A linear neural network model that transforms the desired torque (input layer) into actual torque (output layer) through 1000 neurons (intermediate layer). (**C**) The dots for ***M*** indicate the distribution of the mechanical direction vectors (MDVs) for the 1000 neurons. The dots for ***W*** indicate the distribution of the synaptic weight for the 1000 neurons after learning through error feedback with weight decay. When the distribution of the MDVs is biased toward the 1^st^ and 3^rd^ quadrants, ***W*** converges so that the PD distribution is biased toward the 2^nd^ and 4^th^ quadrants, which is orthogonal to the distribution of the MDVs.

As a simple example of a redundant motor task, we considered a task that requires the production of torque in a two-joint system with redundant actuators (Figure 1A, B). To demonstrate clearly the effect of weight decay, we initially used a simple linear feed-forward neural network that transforms the desired torque (input layer) into actual torque (output layer) through an intermediate layer that consisted of 1000 neurons (Figure 1B). Each neuron in the intermediate layer received a desired torque vector (***τ***) from the input layer with a synaptic weight (***W***) that could be modified with learning. The activation level (***r***) was linearly dependent on the input torque vector (i.e., ***r*** = ***Wτ***), indicating that it obeys cosine tuning. Each neuron generated its own 2D torque vector (mechanical torque direction vector: MDV) that was predetermined by its connection strength (***M***) with the output layer. The total output of the network (***T***) was the vector sum of the output from all neurons. The MDVs were biased toward the first and third quadrants in the torque space (dots for ***M*** in Figure 1C). The network was trained to produce appropriate output torque by randomly presenting 8 target torques (Figure 1A) over 40,000 trials. An error back-propagation algorithm [42] was successively used to modify the synaptic weight (***W***), while the MDV matrix (***M***) was held constant.

**Figure 2:**
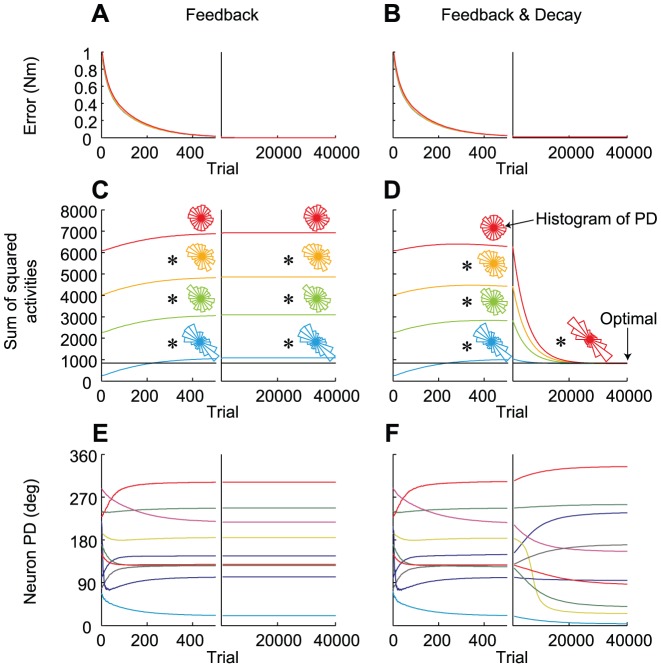
Comparison between the feedback-only and feedback-with-decay rules. Trial-dependent changes in the magnitude of error (**A, B**), the sum of the squared neural activity (**C, D**) averaged across the 8 target conditions, and the PDs of 10 randomly selected neurons (**E, F**), when the synaptic weight was modified with feedback-only (**A, C, E**) or feedback-with-decay rules (**B, D, F**). (**C, D**) The 4 colored lines indicate the changes when various initial synaptic weight conditions were used (see Methods). The distributions of the neuronal PDs at 500^th^ and 40,000^th^ trial for each simulation are shown as polar histograms. The horizontal black line indicates the optimal value calculated analytically as the pseudo-inverse matrix of ***M***.

First, we considered the case where the synaptic weights are solely modified to reduce the error, according to the following equation:
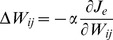
(2)where *α* is the learning rate and *J_e_* is the error cost, as calculated by the error vector (***e*** = ***T***−***τ***) between the output and the desired torque: *J*_e_ = 1/2***e*** ^T^ ***e***. The error gradually decreased and approached zero at around the 500^th^ trial (Figure 2A). Once the error converged to zero, further synaptic modifications did not occur in this model (i.e., the PDs did not change after the 500^th^ trial, Figure 2E), as schematized in Figure S1A. Thus, the cost of the motor effort (the sum of the squared neural activity) did not achieve an optimal level, and the converged states depended on the initial settings for the synaptic weight (Figure 2C; the different colors represent the different initial states). The distribution of the PDs in the converged state also depended on the initial synaptic weight (see polar histograms in Figure 2C). When uncertainty was introduced into the system (i.e., the existence of noise in execution and synaptic modification), the results were almost identical (Figure S2A–C). The synaptic weights randomly moved back and forth along a null trajectory satisfying zero movement error (Figure S2D), which is the natural consequence of redundancy in the motor system [43].

However, the situation was considerably different when modification of the synaptic weights based on error feedback was not perfect, but incorporated *weight decay*, as follows:
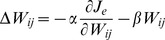
(3)where *β* indicates the decay in motor learning and has a small positive value (*β* = 1.0×10^−4^). In this model, the sum of the squared neural activity converged at an optimal value regardless of the initial synaptic weight (Figure 2D). Importantly, the distribution of the PDs also converged on the same distribution (Figure 2D, * indicates a significant bimodal distribution revealed by the Rayleigh test, *P*<0.05). Why did such a convergence occur? Intuitively, but not mathematically rigorous, this was because, even after error convergence, the synaptic decay term (-*βW_ij_*) continued to induce a very small error. To reduce this small error, the error-feedback term continuously and gradually modified the synaptic weight; as a result, the neuron PDs (Figure 2F) and the distribution of the PDs (polar histogram in Figure 2D) continued to change, until the synaptic weight converged on the optimal state.

In mathematical terms, the modification of the synaptic weights based on the feedback-with-decay rule (Eq. (3)) is similar to the gradient descent rule for minimizing the cost function *J*, which is the weighted sum of the error cost (*J_e_*) and the motor effort cost (*J_m_*):
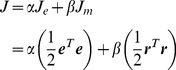
(4)as the gradient descent rule for minimizing *J* is expressed by:
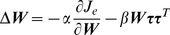
(5)However, it should be noted that Eqs. (3) and (5) do not necessarily minimize the expected value of the cost *J* (i.e., E[*J*]). The reason why we should consider E[*J*] rather than *J* itself is that the optimal solution for the biological system should globally minimize the cost *J* for whole movement directions (see Supporting Text S1). Hereafter, the optimal solution means that it minimizes E[*J*]. In this study, we theoretically proved that the modification rule [Eq. (3)] leads to optimal synaptic weight among many solutions that satisfy zero error under several necessary conditions (see Supporting Text S1): first, the decay rate (*β*) must be much smaller (i.e., slower) than the learning rate (*α*) (condition #1); second, there must be a large number of neurons, each of which generates a quite small output relative to the desired torque magnitude (condition #2); and third, more than two different and independent targets must be practiced (condition #3).

Furthermore, we have also proven that the synaptic weight matrix (***W***) converges to a unique pseudo-inverse of the matrix ***M*** that consists of the MDVs from all of the actuators (see Supporting Text S1).
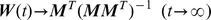
As the synaptic weight matrix determines the PDs of the neurons, the inverse relationship between ***W*** and ***M*** indicates that the distribution of the PD vectors (PDVs) was orthogonal to that of the MDVs. Therefore, when the distribution of the MDVs is biased toward the 1^st^ and 3^rd^ quadrants, the distribution of the converged PDVs should be biased toward the 2^nd^ and 4^th^ quadrants (Figure 1C).

The above results indicate three important points regarding the “feedback-with-decay” rule. First, the optimal solution can be obtained using only *trial-based* error information, without the explicit calculation of the sum of the squared neural activity. Second, the biomechanical properties of the actuators (i.e., MDVs) necessarily determine the neuronal recruitment pattern (i.e., PDVs). Third, the optimal PD bias is steadily formed during the minimization of the motor effort.

Another interesting observation regarding the formation of the bias of the PDs is that when the initial synaptic weight is relatively small (see cyan trace in Figure 2C), even the “feedback-only” rule predicted a PD bias that is similar to the optimal PD bias predicted by the “feedback-with-decay” rule (Figure 2D). By assessing the underlying mechanism mathematically, we found that if a large number of neurons participate in the task (condition #2), the “feedback-only” rule leads the synaptic weight ***W*** to converge on:
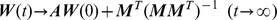
where ***A*** (≠**0**) is a matrix that never increases |***W***(0)| and always satisfies ***MAW***(0) = ***0*** (see Supporting Text S1). This result indicates that if the initial synaptic weight matrix (***W***(0)) is considerably smaller than the pseudo-inverse matrix (***M*** *^T^*(***MM*** *^T^*)^−1^) (condition #4), the converged PD bias is dominated by the PD bias of the pseudo-inverse. Thus, if conditions #2 and #4 are satisfied, even the “feedback-only” rule can predict the approximate direction of the optimal PD bias, even though the converged synaptic weight matrix is not optimal.

In summary, in the linear neural network model, the “feedback-with-decay” rule consistently leads to the optimal synaptic weight and the optimal PD bias, whereas the “feedback-only” rule only predicts the approximate direction of the optimal PD bias in limited conditions.

### Non-linear neural network model with a muscle layer

**Figure 3:**
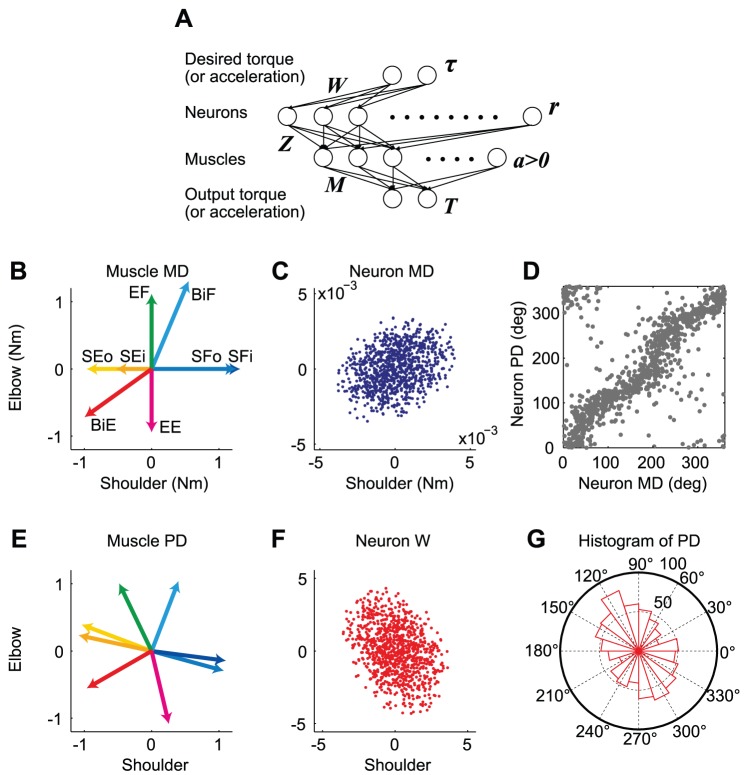
Simulation results for the shoulder and elbow torque exertion task. (**A**) Neural network model with a muscle layer. The model consists of an input layer, a 2^nd^ layer of 1000 neurons, a 3^rd^ layer of 8 muscle groups at the shoulder and elbow joints, and an output layer. (**B**) The mechanical direction vectors (MDVs) for the 8 muscle groups. SFi, inner shoulder flexor (blue); SFo, outer shoulder flexor (light blue); SEi, inner shoulder extensor (orange); SEo, outer shoulder extensor (yellow); EF, elbow flexor (green); EE, elbow extensor (magenta); BiF, biarticular flexor (cyan); and BiE, biarticular extensor (red). (**C**) Distribution of the MDVs for the 1000 neurons in the 2^nd^ layer. (**D**) PDs plotted against MDs. (**E**) PDs for the 8 muscles after learning. (**F**) Distribution of the synaptic weight (i.e., PDVs) for the 1000 neurons after learning. (**G**) Polar histogram of the neuron PDs after learning.

Next, we examined whether these aspects hold true in non-linear neural network models that additionally include a muscle layer whose activity (***a***) was constrained as positive (i.e., muscles do not push) (Figure 3A). Here, it is assumed that the 2^nd^ neural layer consists of corticospinal neurons in M1; however, since M1 actually includes inhibitory interneurons, the layer cannot be regarded as a real M1. Nevertheless, we modeled the neural network incorporating the properties of actual M1 neurons to gain an insight into how the corticospinal neurons are recruited under the feedback-with-decay rule.

Firstly, each corticospinal neuron receives the desired movement parameters from the input layer and their firing rate obeys cosine tuning [44]. Secondly, each corticospinal neuron innervates multiple muscles [45]–[48]. Considering that there are two types of corticospinal neurons [49], one type has direct connections with motoneurons (i.e., cortico-motoneuronal neurons) while the other type indirectly influences motoneurons through spinal interneurons, the innervation weight from the neurons to the muscles (***Z***) is allowed to take positive and negative values. At present, it is assumed that innervation is random and does not have any bias to specific muscles. It is also assumed that the innervation weight (***Z***) is constant through time [50], although this is controversial [51]. These assumptions considerably simplified the model and allowed us to gain a clear insight into the formation of neuronal PDVs relative to the MDVs. Thirdly, the mechanical pulling direction vectors of muscles (***M***) were determined by the muscle parameters (e.g., moment arm) derived from a realistic musculoskeletal model [52], [53]. ***M*** was also kept constant because we only examined the static aspect of movement, e.g., isometric force production or the initial ballistic phase of reaching movements. By simulating these tasks with this network model, we examined whether the feedback-with-decay rule accounts for the reported activation patterns of muscles and M1 neurons.

#### Isometric torque production task

**Figure 4:**
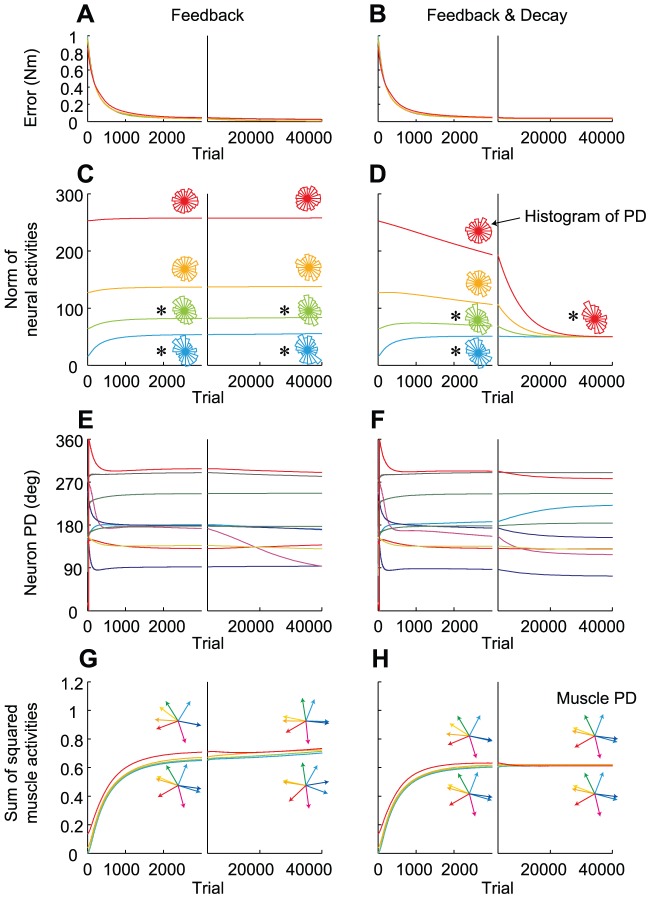
Changes in the error and neural activity for the shoulder and elbow torque exertion task. Trial-dependent changes in the magnitude of error (**A, B**), the norm of the neural activity averaged across the 8 target conditions (**C, D**), the PDs of 10 randomly selected neurons (**E, F**), and the sum of the squared muscle activity averaged across the 8 target conditions (**G, H**), when the synaptic weight was modified with the “feedback-only” (**A, C, E, G**) or “feedback-with-decay” rules (**B, D, F, H**). (**C, D**) The distributions of the neuronal PDs at the 3000^th^ and 40,000^th^ trials for each simulation are shown as polar histograms. (**G, H**) The distributions of the muscle PDs at the 3000^th^ and 40,000^th^ trials for simulations represented by the cyan and red traces in **C** and **D**, respectively, are shown.

First, we simulated the isometric torque production task with a two-joint system (shoulder and elbow) conducted by Herter et al. [2]. The simulation was conducted with 1000 neurons and 8 muscles. Figure 3B shows the MDVs of the muscles in the shoulder-elbow torque plane. Due to the presence of biarticular muscles, the distribution of the MDVs was biased toward the 1^st^ and 3^rd^ quadrants (Figure 3B). The assumption that neurons in the 2^nd^ layer randomly innervate these muscles led to a biased distribution of the neuronal MDVs toward the same quadrants (Figure 3C). We trained the network using the “feedback-only” rule and the “feedback-with-decay” rule, and found that the results were similar to those observed in the linear model. When the decay was incorporated, we found that the motor effort converged on a similar value, irrespective of the initial synaptic weights after the error converged to almost zero (Figure 4B, 4D). In addition, the synaptic weights converged so that the PDs of the neurons in the 2^nd^ layer were bimodally distributed toward the 2^nd^ and 4^th^ quadrants (Figure 3F, 3G, 4D), which was orthogonal to the distribution of the MDs (Figure 3C). In contrast, when the decay was not incorporated, the PD distribution in the converged state depended on the initial synaptic weights (Figure 4C). Thus, our numerical simulation demonstrated that the important points obtained in the linear model were also qualitatively true in the non-linear model. The difference from the linear model was that some neurons change their PDs after the error converged to almost zero under the “feedback-only” rule (Figure 4E), which would be because there was no synaptic weight matrix that strictly satisfies zero error in the case of the non-linear model. The ever-changing PDs somehow contributed to increasing the sum of the squared muscle activity (Figure 4G). The “feedback-with-decay” rule was also advantageous for the suppression of muscle activity (Figure 4H), but the effect was not intense; indeed, even the “feedback-only” rule predicted roughly similar muscle PDs (Figure 4G) as the “feedback-with-decay” rule (Figure 4H). It is also notable that the formation of the PDs of muscles (Figure 4H) was achieved relatively earlier than that of the PD bias of the neurons (Figure 4D) and that the sum of the squared muscle activities were almost the same among all 4 simulations (Figure 4H), irrespective of the large differences in the norm of neural activities (Figure 4D). This may indicate that most of the neural activities cancel each other out at the muscle level to produce similar muscle activation patterns, which is possible because the dimension of neural activity far exceeds that of muscle activity. This may suggest that in such a redundant situation, minimization of neural effort and formation of the optimal PD bias may not be accomplished only by minimizing muscle effort via monitoring the metabolic energy consumed by the muscles.

Interestingly, the predicted PD distribution (Figure 3G) was in agreement with that for M1 neurons in monkeys [2], irrespective of the fact that the corticospinal neurons in our model were only a subset of M1. The bimodal axis of the predicted PD distribution (θ = 121.1°) was within the 99.99% confidence interval of the axis (118.9–158.11°) estimated from the monkeys data [2]. In addition, the resultant vector length (R = 0.162), which represents the strength of the bias, was also within the 99% confidence interval (0.147–0.40). Furthermore, our simulation also predicted the misalignment of muscle MD and PD, which is a key feature of the muscle recruitment pattern [7]–[10], i.e., muscle PDs are located so that they compensate for the sparse part of the MD distribution in the torque space. Thus, even a mono-articular muscle's activation level depends not only on the joint torque but also on the torque of the joint that it does not span (Figure 3E) [8], [9], [54]. Although this misalignment has been considered as a consequence of the minimization of the sum of the squared muscle activity [8]–[11], the feedback-with-decay rule could predict it without an explicit calculation of the sum of the squared muscle activity.

Thus, error-based learning with slight forgetting seems to predict the non-uniform PD distribution of M1 neurons; however, what happens if forgetting is not slight? Theoretical considerations suggest that a relatively larger decay rate led to the system assigning much more weight to minimize the motor effort cost (*J*_m_) than the error cost (*J*_e_). Figure S3 shows the results of simulations conducted with relatively large decay rates that were 5, 10, and 20 times larger than the original *β*. As expected, as the decay rate increased, the motor effort decreased (Figure S3B, S3C) more than necessary to keep the error almost zero, leading to a gradual increase of the converged error level (Figure S3A). Notably, the biased PD distribution gradually disappeared (Figure S3D), clearly indicating that the biased PD distribution emerges as a consequence of effective error-feedback with “slight” forgetting.

#### Initial phase of a reaching movement

**Figure 5:**
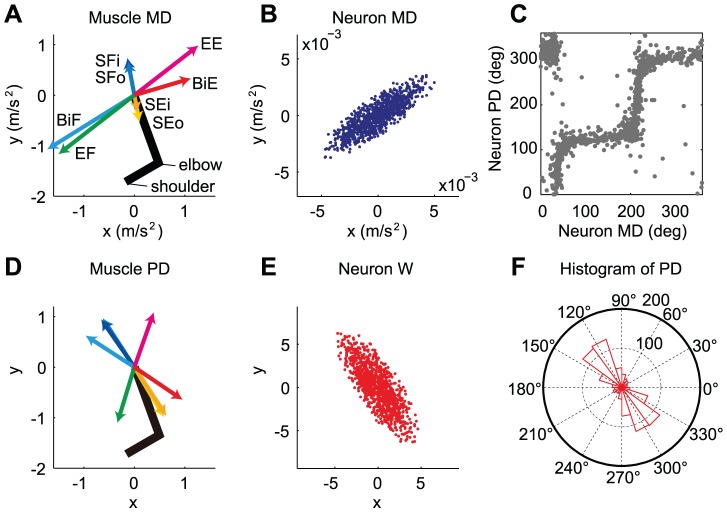
Simulation results for the 2D reaching movements. (**A**) MDVs for the 8 muscle groups. SFi, inner shoulder flexor (blue); SFo, outer shoulder flexor (light blue); SEi, inner shoulder extensor (orange); SEo, outer shoulder extensor (yellow); EF, elbow flexor (green); EE, elbow extensor (magenta); BiF, biarticular flexor (cyan); and BiE, biarticular extensor (red). (**B**) Distribution of the MDVs for 1000 neurons. (**C**) PDs plotted against MDs. (**D**) PDs of the 8 muscles after learning. (**E**) Distribution of the PDVs for 1000 neurons after learning. (**F**) Polar histogram of the neuronal PDs after learning.

**Figure 6:**
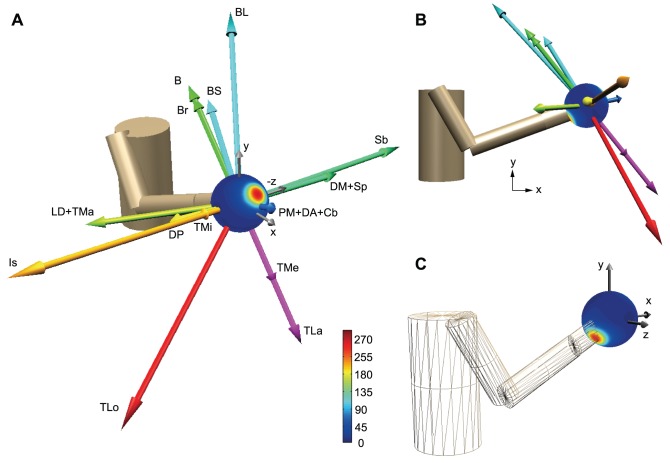
Simulation results for 3D reaching movements. (**A**) The arrows indicate the muscle MDVs in linear acceleration space. DP, posterior part of deltoid; DM, middle part of deltoid; DA, anterior part of deltoid; PM, pectoralis major; Cb, coracobrachialis; LD, latissimus dorsi; TMa, teres major; TMi, teres minor; Is, infraspinatus; Sb, subscapularis; Sp, supraspinatus; BS, short head of the biceps; BL, long head of the biceps; B, brachialis; Br, brachioradialis; TLa, lateral head of the triceps; TMe, medial head of the triceps; and TLo, long head of the triceps. The color gradations indicate the PD density at each point or the number of PDVs within the cone whose semi-angle is 20°. (**B**) Muscle MDVs viewed in the sagittal (x-y) plane. (**C**) PD density viewed from behind the body.

Next, we examined whether the weight decay rule can predict the characteristic bias of the PD distribution of M1 neurons observed during the reaction time period before reaching movements. Since the activity of M1 neurons just before reach initiation would reflect the activity necessary to produce the initial acceleration, we focused on the initial ballistic phase of a reaching movement. To mimic the initial phase, we modified the network by replacing the “desired torque” in Figure 3A with a “desired linear acceleration” of the fingertip in extrinsic space. In this case, the input layer can be considered as the premotor cortex, which represents the desired movement direction in extrinsic space [55], and the muscles can be viewed as linear accelerators of the fingertip (Figure 5A and 6A).

First, we simulated the reaching task with a two-joint system in a horizontal plane described by Scott et al. [3]. The muscle MDVs in a linear acceleration space (i.e., extrinsic space; Figure 5A) was calculated on the basis of the initial limb configuration used in the previous study [27] and the muscle MDVs in the joint torque space (Figure 3B). The MDVs of the muscles and neurons were much more skewed toward the 1^st^ and 3^rd^ quadrants than the torque space (Figure 5A, 5B). We trained the network using the “feedback-only” rule and the “feedback-with-decay” rule, and found that the results were similar to those observed in the linear model. When the decay was incorporated, we found that the distribution of the PDs of the muscles and neurons converged to a much more skewed distribution toward the 2^nd^ and 4^th^ quadrants in linear acceleration space (Figure 5D–F) than that observed in the torque space (Figure 3E–G). These features were in agreement with data from monkeys that were recorded during the reaction time period before reaching [2]. The bimodal axis of the predicted PD distribution (θ = 125.0°) was within the 95% confidence interval of the axis (109.6–127.4°) estimated from the monkeys' data [3]. The resultant vector length (R = 0.507) was within the 99.5% confidence interval (0.19–0.51).

The model was further extended to 3D reaching movements. Figures 6A and 6B show the MDVs for these muscles in linear acceleration space. Although there are strong muscles that accelerate the fingertip toward the right (+z) and left (−z), backward and upward (−x and +y), and forward and downward (+x and −y) directions, there are a few weak muscles that accelerate the fingertip toward the forward and upward (+x and +y) or backward and downward (−x and −y) directions. If neurons randomly innervate these muscles, the neuronal MDVs will have a similar distribution as the muscle MDVs. The network trained with the “feedback-with-decay” rule demonstrated that the distribution of PDVs was enriched in the forward and upward directions (Figure 6A) and in the backward and downward directions (Figure 6C). This is in qualitative agreement with the PDV distribution of M1 neurons recorded in monkeys during the reaction-time period of 3D reaching movements [4]. Although this previous study suggested that the PD distribution of the M1 neurons was supposedly associated with feeding behavior (i.e., the monkeys tended to reach in the forward and upward directions more frequently) [4], our scheme suggests that the PD distribution reflects the biomechanical properties of the musculoskeletal system. To examine whether the PD distribution is influenced by a spatial bias of the reaching direction during learning, we conducted simulations with four different probability conditions for target appearance. In all simulations, the PDs converged to the same distribution that was predicted by the biomechanical properties.

## Discussion

### Trial-by-trial learning with slight forgetting

It has long been hypothesized that well-organized stereotypical movements are achieved by minimizing the cost (*J*), which includes the motor error and the variables related to the motor effort (e.g., jerk, torque change, sum of squared muscle activity, and variance of error) [12]–[15], [23]. It has also been shown that such an optimization model can predict the bias of the PDs of muscles and M1 neurons observed in primate and human experiments [8]–[10], [24], [27]. However, most of the previous optimization studies have examined only the resultant state obtained by the optimization process and largely ignored the underlying trial-by-trial learning process. Therefore, it is unclear how the cost function (*J*) is minimized on a trial-by-trial basis and how the PD biases are formed during optimization.

A small number of previous studies have proposed a mechanism for how the cost of the motor effort is minimized in the brain on a trial-by-trial basis. Kitazawa [26] proposed the “random work hypothesis” in which, in the presence of SDN, the system gradually approaches the optimal state only by successively feeding back trial-based error information. However, there is no guarantee of convergence with the optimal state, especially for highly redundant systems. Indeed, in our highly redundant neural network (n = 1000) with SDN, but without synaptic decay, the synaptic weights were captured at a suboptimal level (Figure S2B). Even when the system was small (n = 2), consistent convergence did not occur (Figure S2D).

In contrast, recent studies have suggested that forgetting might be useful to minimize the motor effort [29]–[35]. Emken et al. [32] demonstrated that trial-by-trial error-feedback learning with forgetting is mathematically equivalent to the minimization of error and effort by formulating the force adaptation task during gait, although their formulation was limited to the case of a single lumped muscle system (i.e., a non-redundant actuator system). An important prediction from this scheme is that the motor system continuously attempts to decrease the level of muscle activation when the movement error is small [30]–[33]. Such a decrease in muscle activity was actually observed when human subjects learned to perform movements in a novel force field environment; initially, muscle activity was increased to reduce the movement error produced by the force perturbation, but once the error decreased to a small value, the muscle activity was gradually decreased [56], [57]. Burdet et al. and Franklin et al. [29], [34], [35] showed that a simple learning rule that incorporates the decay of muscle activity can precisely predict such a specific pattern of change in individual muscle activity during adaptation to various force fields.

The present study further applied the “feedback-with-decay” algorithm to the sensorimotor transformation network, which includes M1 neurons. We initially used a linear neural network and theoretically derived the necessary conditions for convergence on the optimal state. Importantly, these conditions seem to be satisfied in the actual brain. First, the decay rate is known to be much smaller than the learning rate [58]; second, a very large number of M1 neurons actually participate in a single motor task; and third, multiple targets are practiced in real life. Furthermore, using a more realistic non-linear network model, we also confirmed consistent convergence that was irrespective of the initial synaptic weight and spatial bias of the movement directions during practice. These results indicate that weight decay is a more promising process than SDN for a motor system to resolve the redundant actuator problem.

The “feedback-with-decay” rule can be considered as biologically plausible in that it does not need to explicitly calculate the sum of the squared neural activity (total effort cost) by gathering activity information from a vast number of neurons. Since weight decay in each synapse could occur independently of other synapses, a global summation across all neurons would not be needed. Using a framework of weight decay, it would be possible for the CNS to minimize even the motor effort cost during movement of the whole body. One may argue that since we perceive tiredness, the brain must compute the total energetic cost (or motor effort cost); however, to the best of our knowledge, individual neurons that encode the total energetic cost have not been discovered. It is rather likely that such a physical quantity is represented by a large number of distributed neurons in the brain and this distributed information may be perceived as tiredness. Since it is unclear whether the total energetic cost could be readout from such distributed information, decay would be a more promising mechanism for minimizing motor efforts. Furthermore, our simulation results indicate that the formation of an optimal PD distribution pattern for M1 neurons was not necessarily accompanied with the realization of a nearly optimal muscle activation pattern (compare Figure 4D with Figure 4H), suggesting that optimization of motor effort at the neural level could not be accomplished by minimization of muscle effort by monitoring the metabolic energy consumption in the muscles.

Although we referred to the “feedback-with-decay” algorithm as biologically plausible, it should be noted that our simulation algorithm is not fully biologically plausible because it still depends on an artificial calculation (i.e., error back-propagation). Although it is well established that error information is available to the cerebellum [16]–[21], it is unclear how such information is used to modify the activity of individual M1 neurons in the next trial; that is, it is unclear how gradients of error are calculated. Determining a biologically plausible model that does not depend on an artificial calculation remains a major challenge in the field of motor control and learning.

### Stereotypical activity patterns of muscles and M1 neurons

The important point of the present study is that we theoretically proved that the “feedback-with-decay” rule consistently leads the PDs of M1 neurons to converge at a distribution that is orthogonal to the MD distribution. Although Guigon et al. [27] reproduced the skewed PD distribution of M1 neurons for 2D movements, they did not theoretically describe the inverse relationship between the PD and MD distributions, which is probably because they adopted only complex non-linear models and needed to rely only on numerical simulations for solving the optimization problem. In contrast, the present study, which is based on the theoretical background of the linear model, further numerically showed that the inverse relationship also persisted in the non-linear models too.

Importantly, the non-linear model combined with the realistic musculoskeletal parameters can reproduce the non-uniform PD distribution of M1 neurons observed during various motor tasks. The origin of the PD bias has been a hotly debated topic in neurophysiology [59], [60]. Although it has been pointed out that the PD bias observed in 2D postural and reaching tasks emerges as a consequence of the neural compensation of the biomechanical properties [2], [3], the PD bias observed in 3D reaching has been considered to be derived from use-dependent plasticity (i.e., the frequent reaching toward the biased directions accompanying feeding behavior) [4], [5]. One of the reasons for this conflict between the two groups is that they adopted different movement tasks, i.e., one group insisted that 2D tasks with a robotic exoskeleton are advantageous for the comparison of neural activity with accurately measured mechanical variables such as joint motion and joint torque [59], while the other group insisted that unconstraint 3D movements are necessary to reveal the nature of neural activity [60]. The present study is the first to try to resolve this issue. By using a realistic 3D biomechanical model, we found that the PD bias observed in 3D reaching movements by monkeys [4], [5] corresponds to the direction toward which few muscles contribute to the acceleration of the fingertip; the PDs tend to be biased toward the direction according to the weight decay hypothesis. It was also demonstrated that the feedback-with-decay rule always leads the PDs to be biased toward the same direction, irrespective of the spatial bias of the reaching directions during practice. Thus, the weight decay hypothesis suggests that the PD distribution reflects the inverse of the biomechanical properties of the musculoskeletal system (i.e., muscle anatomy and limb configuration). Although it remains to be clarified whether weight decay is actually used for optimization in the brain, the present study provides a unifying framework to understand stereotypical activation patterns of muscles and M1 neurons during 2D and 3D reaching movements.

Another interesting finding is that even the “feedback-only” rule predicts the skewed PD distribution of M1 neurons approximately if the two following conditions are satisfied: a large number of neurons participate in the task (condition #2) and the initial synaptic weight is considerably smaller than the pseudo-inverse matrix (***M*** *^T^*(***MM*** *^T^*)^−1^) (condition #4). This finding indicates that the PD bias itself is not direct evidence of the minimization of effort, as has been thought previously [2], [61]. Nevertheless, we believe that the fact that the optimal PD bias was consistently observed in various motor tasks may reflect the consequence of the minimization of effort because there is no assurance that condition #4 is always satisfied. Thus, theoretically assessing the effects of the error feedback and decay separately, the present study convincingly showed that the decay is essential to reproduce consistently the PD bias observed in the experiments. To verify whether the motor effort is actually minimized and whether weight decay is used during minimization, future studies need to examine the changes in the activity of a large number of neurons for a long period of time.

### Decay must be slight

According to our mathematical consideration, the weight decay rate must be substantially lower than the learning rate (see Supporting Text S1). This necessary condition is biologically very plausible because the strength modulation of the synaptic connections, which is mediated by long-term potentiation and/or long-term depression, is known to decay slowly [58]. It was also demonstrated that, when the decay rate was relatively large, the bias in the PD distribution was not formed and considerable error remained (Figure S3). This clearly indicates that the slightness of the decay is necessary for the formation of the non-uniform PD distribution of M1 neurons.

The present scheme also implies that motor learning has two different time scales: a fast process associated with error correction and a slow process associated with optimizing efficiency through weight decay (Figure 2B, 2D). Due to the coexistence of both time scales, the neural network can assume various unstable states even after motor performance appears to have been achieved [43]; however, after adequate training is conducted to completely learn the task by the slow process, the network should converge to a more stable unique state [62]. The two time scales can be also observed in muscle activity during motor adaptation. While muscle activity rapidly increased in response to the initial large errors caused by a novel perturbation, it was slowly reduced once the error fell below a threshold [34], [56], [57], [63]. The present study suggests that the slow reduction of muscle activity is the result of the optimization process with weight decay. This slow optimization may explain why prolonged training, even after the performance level appears to have reached a plateau, is important [64].

### Limitation of the models and future direction

Due to its simplicity, our model provided clear insights into the role of weight decay on optimization; however, of course, it has several limitations. First, the model considered only corticospinal neurons, although M1 also includes inhibitory interneurons. However, it is noteworthy that our model could predict the PD distribution of M1 neurons recorded from non-human primates, suggesting that most of the neurons recorded in previous experiments were corticospinal neurons. Indeed, considering the large size of corticospinal pyramidal neurons, it is likely that the chance of recording these neurons is relatively high because stable isolation over an extended period of time is required in such experiments [65]. To confirm this possibility, future studies need to examine the PD distribution while distinguishing between interneurons and pyramidal neurons using recently described techniques [66], [67].

Second, a uniform distribution was assumed for the neuron-muscle connectivity (***Z***). As there are no available data for ***Z***, assuming a uniform distribution is reasonable as a first attempt. This assumption results in the distribution of neuron MDs having the same bias as that of muscle MDs. Interestingly, irrespective of such a simple assumption, the model accounted for the PD distribution in various tasks. Since this connectivity depends on the recording site, to resolve this issue, it is necessary to examine the innervation weights of each neuron to the muscles by using a spike triggered average technique as well as the PD of each neuron.

Thirdly, the model only considered static tasks (i.e., isometric force production) and an instantaneous ballistic task (i.e., the initial phase of the reaching movement). Such a single time point model is unrealistic for reaching movements in that it ignores the change of limb posture, posture-dependent changes in the muscle moment arms, multi-joint dynamics during motion, and the deceleration phase. This limitation prevents us from predicting the essential features of movement such as trajectory formation and online trajectory correction [12], [13], [15], [23] that arise from the optimization of a series of motor commands by taking into account the multi-joint dynamics that change according to the limb configuration [68], [69]. However, it is not that our model completely ignores multi-joint dynamics; indeed, we incorporated instantaneous multi-joint dynamics at the initial limb configuration by dealing with the linear acceleration of the fingertip rather than the muscle torque (see Methods). In addition, considering that the CNS does not plan an entire trajectory of movement at the time of movement onset [15], [70], [71], it is likely that the activity of corticospinal neurons just before reach initiation would be largely for the production of the initial acceleration. Thus, the comparison between the neural activity in our model and that recorded during the reaction time period is justified to some extent. However, of course, the present model ignores the effect of events occurring after the initial ballistic phase on the modification of the synaptic weight for the next movement. Finally, the single time point model cannot predict the change of the movement representation in the motor areas that was observed during the course of sensorimotor transformations [72]–[74]. In the future, we need to extend the decay theory to the more dynamic problem of controlling eye or limb movements, including temporal trajectories through motor planning and execution phases [23], [27], [75]–[78]. This dynamic task presents the next major challenge for understanding the neural control of movement.

## Methods

### Linear neural network model

First, we used a linear neural network to transform the desired torque (input layer) into the actual torque (output layer) through an intermediate layer that consisted of 1000 neurons (*n* = 1000) (Figure 1B). Each neuron in the intermediate layer received a desired torque vector (***τ ***∈ ℜ^2^) from the input layer with a synaptic weight (***W*** *_i_* ∈ ℜ^2^) that could be modified with learning. The activation level (*r_i_*) was linearly dependent on the input torque vector (i.e., *r_i_* = ***W*** *_i_^T^* ***τ***), indicating that it obeys cosine tuning [44]. The activation vector for all of the neurons (***r*** ∈ ℜ*^n^*) is expressed as ***r*** = ***Wτ***, where ***W*** ∈ ℜ*^n^* ^×2^ is the synaptic weight matrix for all neurons, expressed as:
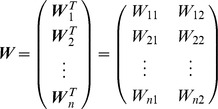
The output vector for each neuron (***T*** *_i_* ∈ ℜ^2^) is determined by its activation level (*r_i_*) multiplied by its mechanical pulling direction vector (MDV) (***M*** *_i_* ∈ ℜ^2^): ***T*** *_i_* = ***M*** *_i_ r_i_*. The total output of the network (***T*** ∈ ℜ^2^) is expressed as the vector sum of the output of all neurons: ***T*** = ***Mr***, where ***M*** ∈ ℜ^2×*n*^ is the matrix of MDVs for all neurons, expressed as:
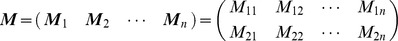
The distribution of the directions of the MDVs was biased toward the first and third quadrants (dots for ***M*** in Figure 1C). ***M*** for the linear model was calculated as ***RM*** *^Uniform8^* ***Z***, where ***R*** = [cos20° sin20°; sin20° cos20°], ***M*** *^Uniform8^* consists of 8 unit vectors that are uniformly distributed in the torque space (e.g., [1 0], [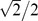 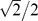], [0 1], etc.), and ***Z*** was the same as the one defined in the non-linear model (see below).

### Learning procedure

The network was trained to produce the appropriate output torque by randomly presenting 8 target torques (Figure 1A) over 40,000 trials. An error back-propagation algorithm [42] was used to successively modify the synaptic weight (***W***), while the MDV matrix (***M***) was kept constant. In order to examine the effect of weight decay, we used 3 learning procedures: 1) feedback-only, 2) feedback-with-noise, and 3) feedback-with-decay rules.

#### 1) Feedback-only rule

In the feedback-only rule, the synaptic weight *W_ij_* was modified by:
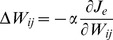
(6)where *α* is the learning rate (*α* = 20) and *J_e_* is the error cost, as calculated by the error vector (***e*** = ***T***−***τ***) between the output torque and desired torque: *J*_e_ = 1/2***e*** ^T^ ***e***.

#### 2) Feedback-with-noise rule

The procedures in the feedback-with-noise rule were the same as in the feedback-only rule, except that SDN was added to the actuator activity and synaptic modification. The activation of each actuator was determined by:
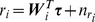
(7)where 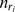 represents white noise with a zero mean and a standard deviation of 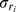, which increased with the magnitude of 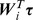 as follows:
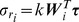
(8)The coefficient of variation *k* was set at 0.25 in the simulation shown in Figure S2. Similarly, the synaptic weight was modified to:
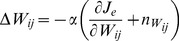
(9)where 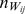 represents white noise with a zero mean and standard deviation 
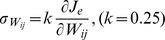
. When *k* = 0.1, the result was the same as that shown in Figure S2, i.e., the synaptic weight converged to a suboptimal solution.

#### 3) Feedback-with-decay rule

In the feedback-with-decay rule, the synaptic weight *W_ij_* was modified by:
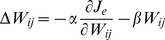
(10)The second term indicates that the change in synaptic weight due to synaptic memory decay in each step is proportional to the current synaptic weight *W_ij_*. This rule is one of the simplest regularization techniques used in machine learning. The decay rate *β* was set to 1.0×10^−4^, much smaller than the learning rate (*α* = 20). We intended to simulate the formation of the PDs of neurons and muscles, which are possibly related to the construction of the synergies and may take a long period of time. Therefore, we used the slowest type of decay time constant (20 days) observed in neurophysiological studies [58], which corresponds to the time constant (τ_β_ = 1/*β* = 10000 trials), assuming that approximately 500 trials are performed in a day. Thus, the current value of *β* (1.0×10^−4^) is much smaller than that of the slow process (4.0×10^−3^) estimated by Smith et al. [79], which is, at most, in the order of hours.

### Initial synaptic weights

The initial synaptic weights were set to random values as follows:
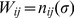
(11)where *n_ij_*(*σ*) represents white noise with a zero mean and standard deviation *σ*. Five different matrices (***W***_1_ *^init^*, ***W***_2_ *^init^*,…, ***W***_5_ *^init^*) were generated using the following 4 standard deviations: *σ* = 0.5 (cyan), 1.5 (green), 2.0 (orange), or 2.5 (red), respectively. These 4 matrices were used for the simulation of the 2 learning rules described above (Figure 2C, 2D) and the feedback-with-noise rule (see Supporting Figure S2B). The MDV matrix ***M*** was also the same for all of the simulations shown in those figures. Therefore, the 3 simulations represented by the same color in those figures were in exactly the same condition at the start of the simulation.

### Non-linear neural network model with a muscle layer

#### Intrinsic torque space model (2D)

To confirm the effectiveness of weight decay in a more realistic model, we also considered a neural network model with a muscle layer whose activity (***a***) was constrained to be positive (i.e., the muscles did not push) (Figure 3A); the muscle layer consisted of the 8 muscles at the shoulder and elbow joints (Figure 3B). We assumed that the neurons in the model directly activate the muscles, i.e., we assumed that the neural layer consisted of only corticospinal neurons. The neurons received the desired movement parameters from the input layer and their firing rate obeyed cosine tuning [44]. On the basis of anatomical and electrophysiological findings, we assumed that each neuron innervates multiple muscles [45]–[48]. The innervation weights for each neuron to the 8 muscles [***Z*** *_i_* = (*Z*_1*i*_ *Z*_2*i*_… *Z*_8*i*_)*^T^*] were established so that ***Z*** *_i_*s (*i* = 1–1000) were uniformly distributed on the surface of a sphere in 8-dimensional space, the radius of which was 0.002 (2/*n*). The activation of each muscle (*a_i_*) was expressed as the sum of the effects from all of the neurons:
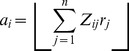
(12)where the operator ⌊ ⌋ indicates that ⌊*x*⌋ = *x* for *x*>0 and ⌊*x*⌋ = 0 for *x*≤0. The total output of the network (***T***) is expressed as the vector sum of the output from all of the muscles: ***T = M*** *^In^* ***a***, where ***a*** = (*a*_1_ *a*_2_…*a*_8_)*^T^* is the activation vector for all of the muscles and ***M*** *^In^* = (***M***_1_ *^In^* ***M***_2_ *^In^*…***M***_8_ *^In^*) ∈ ℜ^2×8^ is a matrix that consists of the MDVs for all of the muscles.

Using realistic muscle data, we modeled a 2D upper limb that had 2 degrees of freedom (DOF; shoulder and elbow joints) with 26 muscle elements (Table S2). For the physiological cross-sectional areas (PCSA) and pennation angles (Table S2), we used published data from *Macaca mulatta* [52]. For the moment arms of the muscles, we extracted data from a human musculoskeletal model [53] in which the shoulder was abducted at 90° and horizontally flexed at 30°, and the elbow was flexed at 90°. The MDV for each muscle in intrinsic space was calculated as:
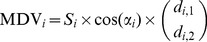
(13)where *S_i_* is the PCSA, *α_i_* is the pennation angle, and (*d_i_*_,1_ *d_i_*_,2_)*^T^* are the moment arms for the shoulder and elbow joints. Assuming that muscles with similar mechanical properties should behave in a similar fashion, we grouped the 26 muscle elements into 8 groups. Thus, the MDV matrix for the 8 muscle groups in intrinsic space is obtained as ***M*** *^In^* ∈ ℜ^2×8^. We defined the effect of the activation of each neuron on the output torque (***M*** *^In^* ***Z*** *_i_*) as the “*MDV of neuron i*.” The matrix of MDVs for all of the neurons was defined as ***M*** *^In^* ***Z***, where ***Z*** = (***Z***_1_ ***Z***_2_…***Z*** *_n_*) ∈ ℜ^8×*n*^. To examine the effects of the initial conditions, the simulation was conducted 4 times with different sets of initial synaptic weights [*σ* = 0.5 (cyan), 2.0 (green), 4.0 (orange), or 8.0 (red) in Eq. (11)].

#### Extrinsic space model (2D)

The network model can also be applied to the task of producing the linear acceleration of the fingertip (i.e., the initial phase of the reaching movement) by replacing the torque in Figure 3A with a linear acceleration of the fingertip in extrinsic space. In this case, the input layer can be considered as the premotor cortex that represents the desired movement direction in extrinsic space [55] and the muscles can be viewed as linear accelerators of the fingertip (see Figure 5A and 6A). The MDV for each muscle in linear acceleration space (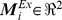) was calculated as:
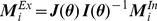
(14)where ***J***(***θ***) ∈ ℜ^2×2^ is the Jacobian matrix, ***I***(***θ***) ∈ ℜ^2×2^ is the system inertia matrix of the two-joint system, and ***θ*** ∈ ℜ^2^ is a joint angle vector that consists of the shoulder and elbow angles. To calculate the Jacobian and inertia matrices, we used morphological data from *M. mulatta* (Table S1). We used the MDV matrix for 8 muscles in extrinsic space expressed by:
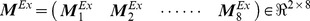
(15)as ***M*** in Figure 3A for the simulation of 2D reaching movements (Figure 5). To examine the effects of the initial conditions, the simulation was conducted 4 times with different sets of initial synaptic weights [*σ* = 0.5 (cyan), 2.0 (green), 4.0 (orange), or 8.0 (red) in Eq. (11)].

#### Extrinsic space model (3D)

We further extended the model to 3D reaching movements. We modeled a 3D upper limb with 4 DOF; (3 DOF for the shoulder and 1 DOF for the elbow) with 26 muscle elements (Table S3). In order to match the initial limb posture in our simulation with that of the 3D reaching movements used in previous primate studies [4], [5], we set the shoulder flexion angle at 30°, the internally rotated shoulder angle at 12°, and the elbow flexion angle at 80°. In this posture, the fingertip position was at shoulder level in the midsagittal plane (Figure 6). Moment arms in this posture were extracted from a human musculoskeletal model [53] and are listed in Table S3. The MDV for each muscle in intrinsic space was calculated as:
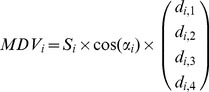
(16)where *S_i_* is the PCSA, *α_i_* is the pennation angle, and (*d_i_*_,1_ *d_i_*_,2_ *d_i_*_,3_ *d_i_*_,4_)*^T^* are the moment arms for the 3 DOF at the shoulder (**x_U_**, **y_U_**, and **z_U_** in Figure S4A) and 1 DOF at the elbow (**z_F_**). Assuming that muscles with similar mechanical properties should behave in a similar fashion, we grouped the 26 muscle elements into 16 groups. Therefore, the muscle MDV matrix in intrinsic space is a 4×16 matrix, as follows:
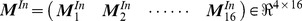
(17)The Jacobian matrix ***J***(***θ***) ∈ ℜ^3×4^ and the system inertia matrix ***I***(***θ***)∈ℜ^4×4^ for the 3D limb model were calculated using previously described methods [80]. Note that, in the present study, we used a segment-fixed coordinate (Figure 2B in [80]) as the generalized coordinate, although the joint coordinate (Figure 2C in [80]) was used in the previous study. The linear acceleration of the fingertip produced by each muscle (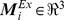) was calculated as:
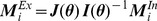
(18)We used the matrix of MDVs for 16 muscles in extrinsic space expressed by:
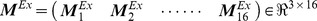
(19)as ***M*** for the simulation of 3D reaching movements.

For the 3D simulation, 14 equally spaced targets (Figure S4B) were randomly presented over 100,000 trials. The learning rate (*α*) and forgetting rate (*β*) were set to 500 and 1.0×10^−4^, respectively. To examine the effects of the initial conditions, the simulation was conducted 5 times with different sets of initial synaptic weights [*σ* = 0.9, 1.8, 2.7, 3.6, or 4.5 in Eq. (11)]. We also examined the effect of the spatial bias of reaching movements during learning (i.e., non-uniform probability of target appearance) using the following 4 conditions:

1. the probability of appearance was equal for all 14 targets;
2. the probability for targets #1 and #3 was 8/28 (1/28 for the other targets);
3. the probability for targets #2 and #4 was 8/28 (1/28 for the other targets);
4. the probability for targets #5 and #6 was 8/28 (1/28 for the other targets).

In total, we conducted 20 (5 initial weights×4 probability conditions) simulations.

### Analysis of the PD distribution

To examine the significance of the bimodal distribution obtained from the simulation, we performed the Rayleigh test for uniformity against a bimodal alternative (*P*<0.05) using a circular statistics toolbox [81]. To quantify the characteristics of the PD distribution, 2 parameters were calculated for each PD distribution. We multiplied the PDs by 2, transformed them to unit vectors in the 2D plane using 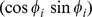, and took a vector summation across all PDs to obtain the resultant vector. The direction (θ) and length (R) of the resultant vector represents the direction and strength of the PD bias, respectively. To compare them with experimental data, we extracted the raw PD data from the literature (178 neurons for a 2D isometric task from Figure 9A in [2] and 141 neurons for a 2D reaching task from Figure 3B in [3]) and estimated the confidence intervals for both parameters using a bootstrapping procedure with 10000 times resampling.

## Supporting Information

Figure S1 **Comparison between the feedback-only and feedback-with-decay rules using a simple redundant problem.** To graphically illustrate the behavior of synaptic weight in the two modification rules, we simulated a simple redundant problem to find the set of w_1_ and w_2_ that fulfills the equation: w_2_−w_1_ = 1. The color gradations indicate the error cost as a function of the synaptic weights w_1_ and w_2_. The white dashed line indicates the minimum at which the error is zero. The circles indicate the contours of the sum of the squared values (i.e., w_1_^2^+w_2_^2^). Simulations were conducted with w_1_ = 0 and w_2_ = −2 as the initial values. In the feedback-only rule (**A**), modification ceased after the error reached zero, whereas in the feedback-with-decay rule (**B**), modification continued after the error reached zero and the sum of the squared values had converged with the minimum value.
(TIF)

Figure S2 **Simulation results by the feedback-with-noise rule.** (**A–C**) Simulation results for the neural network model shown in Figure 1, using the feedback-with-noise rule. Trial-dependent changes in the magnitude of error (**A**), the sum of the squared neural activity (**B**) averaged across the 8 target conditions, and the PDs of 10 randomly selected neurons (**C**). (**D**) Simulations of a simple redundant system to find the set of w_1_ and w_2_ that fulfills the equation: w_2_−w_1_ = 1 using the feedback-with-noise rule.
(TIF)

Figure S3 **Simulation results for the torque exertion task with relatively large weight decay rates.** (**A–C**) Trial-dependent changes in the magnitude of the error (**A**), the norm of the neural activity averaged across the 8 target conditions (**B**), and the sum of the squared muscle activity averaged across the 8 target conditions (**C**), when the synaptic weight was modified by error feedback with relatively large decay rates that were 5, 10, and 20 times larger than the original *β* (Figure 4). The 4 colored lines indicate the changes when various initial synaptic weight conditions were used. (**D**) Distribution of the synaptic weight (i.e., PDVs) for the 1000 neurons after learning.
(TIF)

Figure S4 **Model and motor task for 3D reaching movements.** (**A**) Segment-fixed coordinate systems for the upper arm segment (**x_U_**, **y_U_**, **z_U_**) and forearm-and-hand segment (**x_F_**, **y_F_**, **z_F_**). (**B**) The 14 equally spaced targets used for the simulation of 3D reaching movements.
(TIF)

Table S1 **Parameters for the segments.** p is the position of the center of mass measured from the proximal joint, represented as a % of the segment length. kx, ky, and kz are the radii of gyration about the x, y, and z axes of the segment, respectively, represented as a % of the segment length. These data are for the *Macaca Mulatta* (6 kg) [52], except that ky was taken from human male data [82].
(DOC)

Table S2 **Parameters for the muscles in the horizontal plane in the 2-DOF upper extremity model.** *S* is the physiological cross-sectional area. *α* is the pennation angle. *d*_s_ and *d*_e_ are the moment arms for shoulder flexion(+)/extension(−) and elbow flexion(+)/extension(−), respectively.
(DOC)

Table S3 **Moment arms for the muscles in the 4-DOF model in 3D space.** *d*_1_, *d*_2_, and *d*_3_ are the shoulder joint moment arms for the **x_U_**, **y_U_**, and **z_U_** axes, respectively. *d*_4_ is the elbow joint moment arm for the **z_F_** axis.
(DOC)

Text S1 **Mathematical derivations.** This document provides 1) mathematical derivation of the optimal synaptic weights and 2) mathematical proof of convergence for the linear neural network model of sensorimotor transformation.
(PDF)
