# Supplementary figures and images for: Learning with Slight Forgetting Optimizes Sensorimotor Transformation in Redundant Motor Systems
Source: PLoS Comput Biol. 2012 Jun 28;8(6):e1002590. doi: 10.1371/journal.pcbi.1002590 (PMC3386159; doi:10.1371/journal.pcbi.1002590)

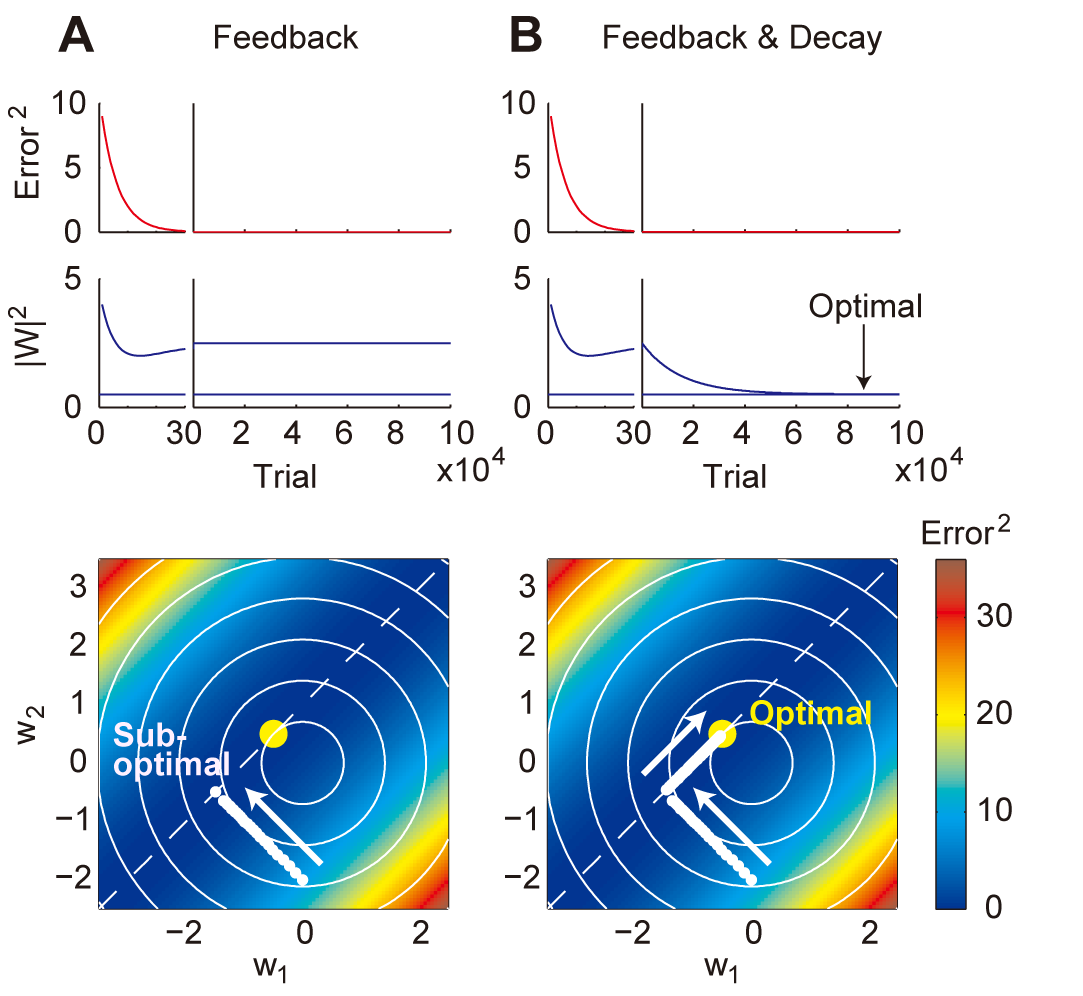

Supplement: Figure S1 — Comparison between the feedback-only and feedback-with-decay rules using a simple redundant problem. To graphically illustrate the behavior of synaptic weight in the two modification rules, we simulated a simple redundant problem to find the set of w1 and w2 that fulfills the equation: w2−w1 = 1. The color gradations indicate the error cost as a function of the synaptic weights w1 and w2. The white dashed line indicates the minimum at which the error is zero. The circles indicate the contours of the sum of the squared values (i.e., w1 2+w2 2). Simulations were conducted with w1 = 0 and w2 = −2 as the initial values. In the feedback-only rule (A), modification ceased after the error reached zero, whereas in the feedback-with-decay rule (B), modification continued after the error reached zero and the sum of the squared values had converged with the minimum value. (TIF) [file pcbi.1002590.s001.tif]

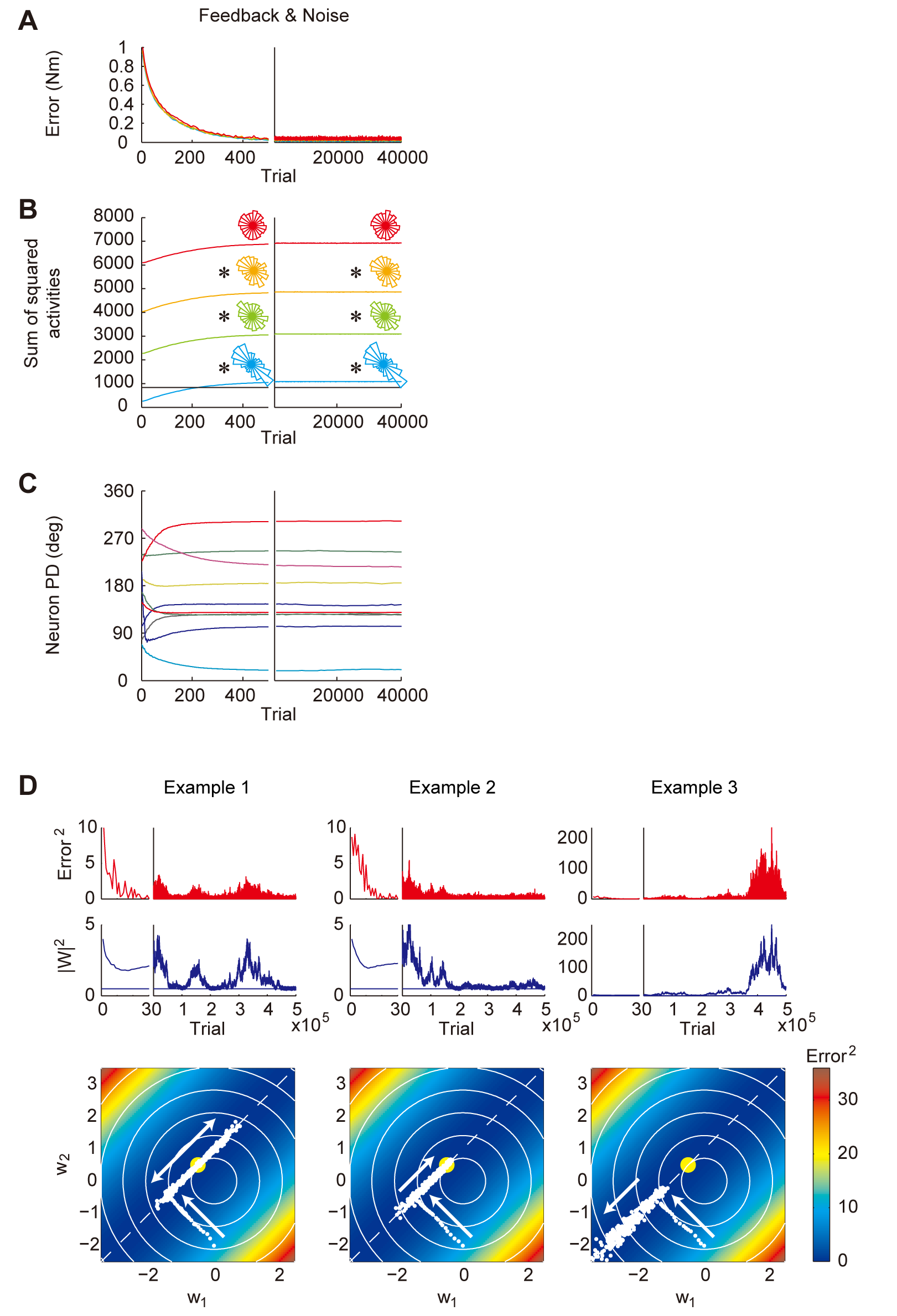

Supplement: Figure S2 — Simulation results by the feedback-with-noise rule. (A–C) Simulation results for the neural network model shown in Figure 1, using the feedback-with-noise rule. Trial-dependent changes in the magnitude of error (A), the sum of the squared neural activity (B) averaged across the 8 target conditions, and the PDs of 10 randomly selected neurons (C). (D) Simulations of a simple redundant system to find the set of w1 and w2 that fulfills the equation: w2−w1 = 1 using the feedback-with-noise rule. (TIF) [file pcbi.1002590.s002.tif]

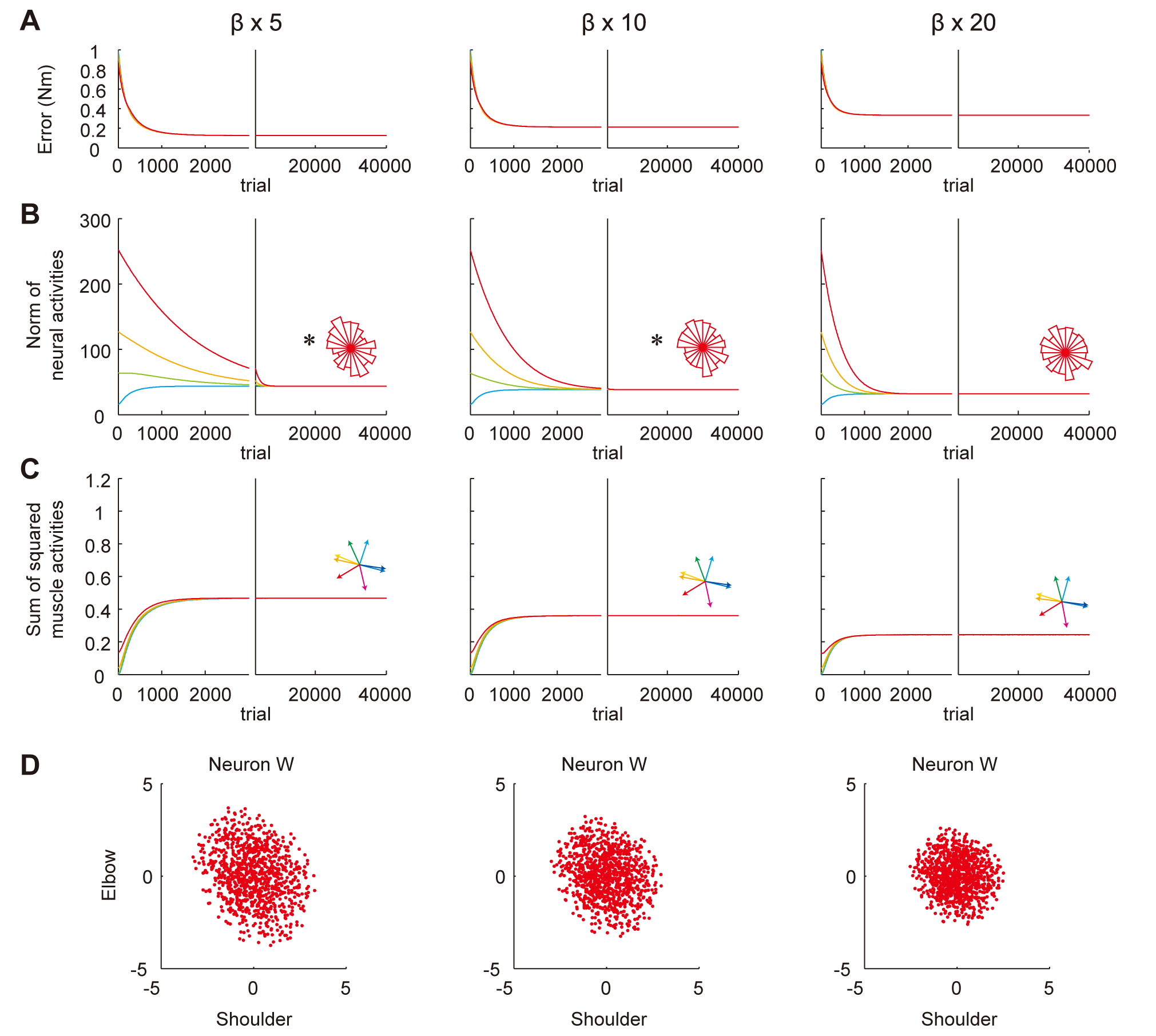

Supplement: Figure S3 — Simulation results for the torque exertion task with relatively large weight decay rates. (A–C) Trial-dependent changes in the magnitude of the error (A), the norm of the neural activity averaged across the 8 target conditions (B), and the sum of the squared muscle activity averaged across the 8 target conditions (C), when the synaptic weight was modified by error feedback with relatively large decay rates that were 5, 10, and 20 times larger than the original β (Figure 4). The 4 colored lines indicate the changes when various initial synaptic weight conditions were used. (D) Distribution of the synaptic weight (i.e., PDVs) for the 1000 neurons after learning. (TIF) [file pcbi.1002590.s003.tif]

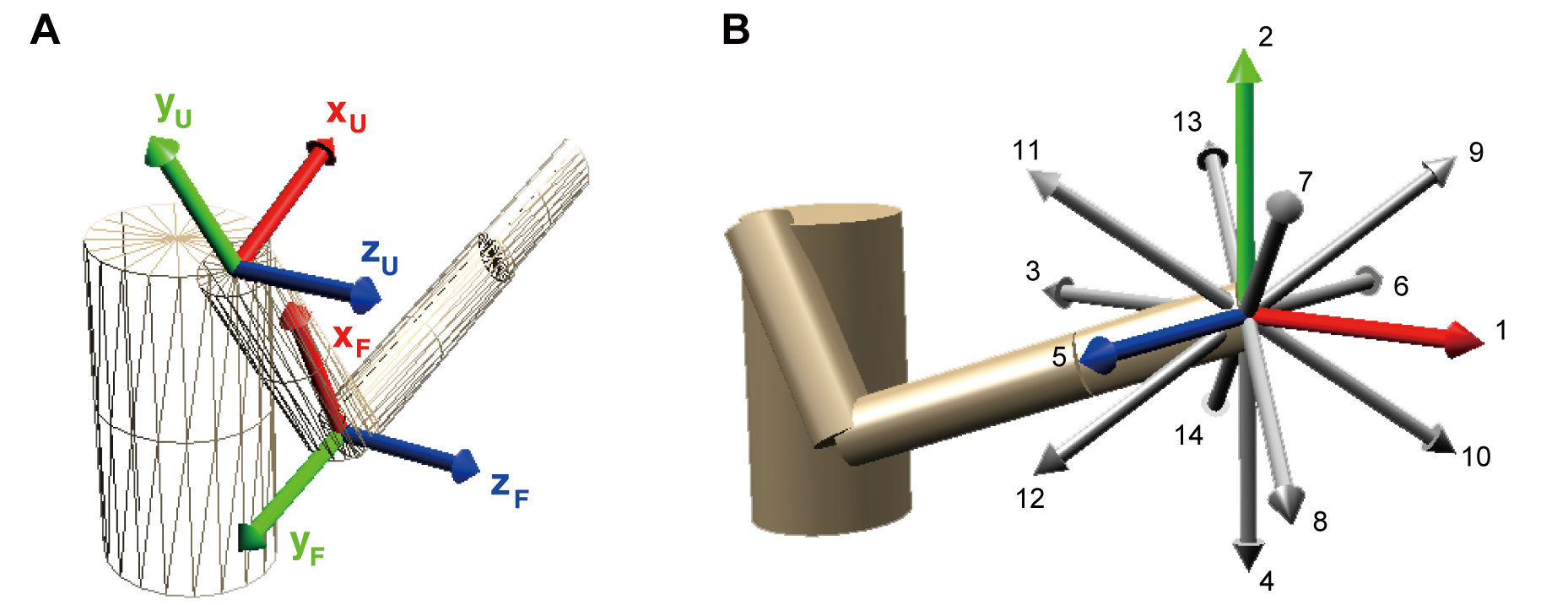

Supplement: Figure S4 — Model and motor task for 3D reaching movements. (A) Segment-fixed coordinate systems for the upper arm segment (xU, yU, zU) and forearm-and-hand segment (xF, yF, zF). (B) The 14 equally spaced targets used for the simulation of 3D reaching movements. (TIF) [file pcbi.1002590.s004.tif]
