# Supplementary material for: Learning with Slight Forgetting Optimizes Sensorimotor Transformation in Redundant Motor Systems
Source: PLoS Comput Biol. 2012 Jun 28;8(6):e1002590. doi: 10.1371/journal.pcbi.1002590 (PMC3386159; doi:10.1371/journal.pcbi.1002590)
Supplement: Table S1 — Parameters for the segments. p is the position of the center of mass measured from the proximal joint, represented as a % of the segment length. kx, ky, and kz are the radii of gyration about the x, y, and z axes of the segment, respectively, represented as a % of the segment length. These data are for the Macaca Mulatta (6 kg) [52], except that ky was taken from human male data [82]. (DOC) [file pcbi.1002590.s005.doc]

**Table S1. Parameters for the segments.**

| Segment | Mass (g) | Length (m) | p (%) | kx (%) | ky (%) | kz (%) |
| --- | --- | --- | --- | --- | --- | --- |
| Upper arm | 228 | 0.145 | 50 | 24.7 | 15.8 | 24.7 |
| Forearm | 150 | 0.17 | 44 | 26 | 12.1 | 26 |
| Hand | 48 | 0.104 | 39 | 24.8 | 18.4 | 24.8 |
