# Supplementary material for: Learning with Slight Forgetting Optimizes Sensorimotor Transformation in Redundant Motor Systems
Source: PLoS Comput Biol. 2012 Jun 28;8(6):e1002590. doi: 10.1371/journal.pcbi.1002590 (PMC3386159; doi:10.1371/journal.pcbi.1002590)
Supplement: Table S2 — Parameters for the muscles in the horizontal plane in the 2-DOF upper extremity model. S is the physiological cross-sectional area. α is the pennation angle. d s and d e are the moment arms for shoulder flexion(+)/extension(−) and elbow flexion(+)/extension(−), respectively. (DOC) [file pcbi.1002590.s006.doc]

**Table S2. Parameters for the muscles in the horizontal plane in the 2-DOF upper extremity model.**

| Muscle | Group | *S* (cm^2^) | *α* (deg) | *d*_s_ (cm) | *d*_e_ (cm) |
| --- | --- | --- | --- | --- | --- |
| Deltoid posterior (DP) | SEo | 2 | ― | -3.73 | ― |
| Deltoid middle (DM) | SEo | 5.2 | 20 | -0.24 | ― |
| Deltoid anterior (DA) | SFo | 1.9 | ― | 2.76 | ― |
| Pectoralis major 1 (PM1) | SFo | 1.52 | ― | 4.65 | ― |
| Pectoralis major 2 (PM2) | SFo | 1.52 | ― | 3.70 | ― |
| Pectoralis major 3 (PM3) | SFo | 1.52 | ― | 2.88 | ― |
| Pectoralis major 4 (PM4) | SFo | 1.52 | ― | 2.54 | ― |
| Pectoralis major 5 (PM5) | SFo | 1.52 | ― | 1.88 | ― |
| Latissimus dorsi 1 (LD1) | SEo | 0.94 | ― | -2.24 | ― |
| Latissimus dorsi 2 (LD2) | SEo | 0.94 | ― | -2.19 | ― |
| Latissimus dorsi 3 (LD3) | SEo | 0.94 | ― | -2.05 | ― |
| Latissimus dorsi 4 (LD4) | SEo | 0.94 | ― | -1.81 | ― |
| Latissimus dorsi 5 (LD5) | SEo | 0.94 | ― | -1.71 | ― |
| Coracobrachialis (Cb) | SFi | 1.4 | 13 | 2.17 | ― |
| Infraspinatus (Is) | SEi | 8.6 | 15 | -1.04 | ― |
| Subscapularis (Sb) | SFi | 15.8 | 22 | 1.54 | ― |
| Supraspinatus (Sp) | SFi | 6.9 | 15 | 1.11 | ― |
| Teres major (TMa) | SEo | 3.7 | 14 | -1.01 | ― |
| Teres minor (TMi) | SEi | 1.5 | 12 | -1.36 | ― |
| Biceps short (BS) | BiF | 2.6 | ― | 1.99 | 4.38 |
| Biceps long (BL) | BiF | 4.6 | 7 | 1.76 | 4.38 |
| Brachialis (B) | EF | 3.7 | ― | ― | 3.25 |
| Brachioradialis (Br) | EF | 1.7 | ― | ― | 7.89 |
| Triceps lateral (TLa) | EE | 7.7 | 21 | ― | -1.85 |
| Triceps medial (TMe) | EE | 4.4 | 18 | ― | -1.85 |
| Triceps long (TLo) | BiE | 10.4 | 31 | -2.57 | -1.85 |
