# Supplementary material for: Learning with Slight Forgetting Optimizes Sensorimotor Transformation in Redundant Motor Systems
Source: PLoS Comput Biol. 2012 Jun 28;8(6):e1002590. doi: 10.1371/journal.pcbi.1002590 (PMC3386159; doi:10.1371/journal.pcbi.1002590)
Supplement: Table S3 — Moment arms for the muscles in the 4-DOF model in 3D space. d 1, d 2, and d 3 are the shoulder joint moment arms for the xU, yU, and zU axes, respectively. d 4 is the elbow joint moment arm for the zF axis. (DOC) [file pcbi.1002590.s007.doc]

**Table S3. Moment arms for the muscles in the 4-DOF model in 3D space.**

| Muscle | Group | *d*_1_ (cm) | *d*_2_ (cm) | *d*_3_ (cm) | *d*_4_ (cm) |
| --- | --- | --- | --- | --- | --- |
| Deltoid posterior (DP) | ― | -0.06 | -2.97 | -2.29 | ― |
| Deltoid middle (DM) | ― | -2.47 | 0.30 | 0.20 | ― |
| Deltoid anterior (DA) | ― | 2.36 | 1.31 | 2.34 | ― |
| Pectoralis major 1 (PM1) | PM | 4.81 | 1.50 | 4.01 | ― |
| Pectoralis major 2 (PM2) | PM | 5.42 | 1.51 | 2.02 | ― |
| Pectoralis major 3 (PM3) | PM | 4.69 | 1.30 | 0.32 | ― |
| Pectoralis major 4 (PM4) | PM | 4.53 | 0.88 | 0.06 | ― |
| Pectoralis major 5 (PM5) | PM | 3.89 | 0.60 | -0.69 | ― |
| Latissimus dorsi 1 (LD1) | LD | 1.95 | -0.78 | -3.59 | ― |
| Latissimus dorsi 2 (LD2) | LD | 1.79 | -0.72 | -3.68 | ― |
| Latissimus dorsi 3 (LD3) | LD | 1.78 | -0.72 | -3.57 | ― |
| Latissimus dorsi 4 (LD4) | LD | 1.74 | -0.70 | -3.31 | ― |
| Latissimus dorsi 5 (LD5) | LD | 1.44 | -0.59 | -3.21 | ― |
| Coracobrachialis (Cb) | ― | 0.46 | 0.11 | 2.18 | ― |
| Infraspinatus (Is) | ― | -0.16 | -2.36 | -0.34 | ― |
| Subscapularis (Sb) | ― | 0.70 | 1.58 | -0.17 | ― |
| Supraspinatus (Sp) | ― | -1.42 | 0.38 | 0.38 | ― |
| Teres major (TMa) | ― | 3.80 | 0.06 | -3.23 | ― |
| Teres minor (TMi) | ― | 0.90 | -2.31 | -0.75 | ― |
| Biceps short (BS) | Bi | 0.42 | 0.22 | 1.98 | 4.03 |
| Biceps long (BL) | Bi | -0.62 | 1.31 | 0.42 | 4.04 |
| Brachialis (B) | ― | ― | ― | ― | 2.93 |
| Brachioradialis (Br) | ― | ― | ― | ― | 7.04 |
| Triceps lateral (TLa) | Tri | ― | ― | ― | -1.94 |
| Triceps medial (TMe) | Tri | ― | ― | ― | -1.94 |
| Triceps long (TLo) | ― | 2.59 | -0.76 | -2.80 | -1.94 |
