# Supplementary material for: Learning with Slight Forgetting Optimizes Sensorimotor Transformation in Redundant Motor Systems
Source: PLoS Comput Biol. 2012 Jun 28;8(6):e1002590. doi: 10.1371/journal.pcbi.1002590 (PMC3386159; doi:10.1371/journal.pcbi.1002590)
Supplement: Text S1 — Mathematical derivations. This document provides 1) mathematical derivation of the optimal synaptic weights and 2) mathematical proof of convergence for the linear neural network model of sensorimotor transformation. (PDF) [file pcbi.1002590.s008.pdf]

Learning with slight forgetting optimizes sensorimotor transformation in redundant motor systems

Masaya Hirashima, Daichi Nozaki

Graduate School of Education, The University of Tokyo,
Bunkyo-ku, Tokyo 113-0033, Japan

1. Optimal synaptic weights

To find the optimal synaptic weights, we must define the criteria for optimality (i.e., the cost function). It is reasonable to assume that the optimal synaptic weights would minimize the movement error and the motor effort, wherever a target appears in the task space. Mathematically, the aim is to find the synaptic weights that minimize the expected value of the weighted sum of the error cost and the motor effort cost:

$$\begin{aligned} E\left[J\right] &= E\left[\alpha J_e + \beta J_m\right] \\ &= E\left[\alpha\left(\frac{1}{2}\boldsymbol{e}^T\boldsymbol{e}\right) + \beta\left(\frac{1}{2}\boldsymbol{r}^T\boldsymbol{r}\right)\right] \end{aligned} \quad \text{(A1)}$$

with the condition that the mean ($\boldsymbol{m}$) and covariance matrix ($\boldsymbol{C}$) of target $\boldsymbol{\tau}$ are:

$$\begin{cases} \boldsymbol{m} = \begin{pmatrix} 0 & 0 \end{pmatrix}^T \\ \boldsymbol{C} = c\begin{pmatrix} 1 & 0 \\ 0 & 1 \end{pmatrix} \end{cases} \quad \text{(A2)}$$

Because the error cost (J_e) can be represented as $J_e = ½(\boldsymbol{B\tau})^T\boldsymbol{B\tau}$ using $\boldsymbol{B} = \boldsymbol{MW} - \boldsymbol{I} \in \Re^{2\times2}$, the expected value of the error cost can be written as:

$$\begin{aligned} E\left[J_e\right] &= E\left[\frac{1}{2}\boldsymbol{e}^T\boldsymbol{e}\right] = E\left[\frac{1}{2}(\boldsymbol{B\tau})^T\boldsymbol{B\tau}\right] \\ &= \frac{1}{2}\left(Tr(\boldsymbol{BCB}^T) + (\boldsymbol{Bm})^T\boldsymbol{Bm}\right) \\ &= \frac{1}{2}Tr(\boldsymbol{BCB}^T) \\ &= \frac{c}{2}(B_{11}{}^2 + B_{12}{}^2 + B_{21}{}^2 + B_{22}{}^2) \end{aligned} \quad \text{(A3)}$$

The expected value of the motor effort cost (J_m) can be written as:

$$\begin{aligned}E[J_m] &= E\left[\frac{1}{2}\boldsymbol{r}^T\boldsymbol{r}\right] = E\left[\frac{1}{2}(\boldsymbol{W\tau})^T\boldsymbol{W\tau}\right] \\ &= \frac{1}{2}\left(Tr(\boldsymbol{WCW}^T) + (\boldsymbol{Wm})^T\boldsymbol{Wm}\right) \\ &= \frac{1}{2}Tr(\boldsymbol{WCW}^T) \\ &= \frac{c}{2}\left(\sum W_{i1}{}^2 + \sum W_{i2}{}^2\right) \\ &= \frac{c}{2}\left(\sum_{ij} W_{ij}{}^2\right)\end{aligned} \tag{A4}$$

It is now apparent that the Moore-Penrose pseudoinverse ($\boldsymbol{M}^+ \in \Re^{n\times 2}$) of $\boldsymbol{M}$ is the optimal solution that minimizes the cost function $E[J]$ among the many solutions satisfying zero error, for the following two reasons. First, since $\boldsymbol{B} = \boldsymbol{MW} - \boldsymbol{I} = \boldsymbol{MM}^+ - \boldsymbol{I} = 0$, $E[J_e]$ becomes 0. Second, the 1st column vector ($\boldsymbol{M}^+_{(1)}$) of $\boldsymbol{M}^+$ is the solution with the smallest Euclidean norm among the many solutions of $\boldsymbol{W}_{(1)} \in \Re^{n\times 1}$ that satisfy the equation $\boldsymbol{MW}_{(1)} = \begin{pmatrix}1\\0\end{pmatrix}$, and the 2nd column vector ($\boldsymbol{M}^+_{(2)}$) of $\boldsymbol{M}^+$ is the solution with the smallest Euclidean norm among the many solutions of $\boldsymbol{W}_{(2)} \in \Re^{n\times 1}$ that satisfy the equation $\boldsymbol{MW}_{(2)} = \begin{pmatrix}0\\1\end{pmatrix}$. Therefore, $E[J_m]$ is also minimized, because $E[J_m]$ is represented as the sum of squares of the elements of the two vectors $\boldsymbol{W}_{(1)}$ and $\boldsymbol{W}_{(2)}$ in Eq. (A4).

2. Mathematical proof of convergence

In this section, we prove that the synaptic weight matrix $\boldsymbol{W}$ converges to the pseudoinverse of the matrix $\boldsymbol{M}$ if the synaptic weights are modified by the feedback-with-decay rule (Eq. (3) in the main text) in the linear neural network model (Figure 1B). $\boldsymbol{W}$ at the $(t+1)^{\text{th}}$ trial can be written as:

$$\begin{aligned} \boldsymbol{W}(t+1) &= \boldsymbol{W}(t) + \Delta \boldsymbol{W}(t) \\ &= \boldsymbol{W}(t) + \left(-\alpha \frac{\partial J_e}{\partial \boldsymbol{W}(t)} - \beta \boldsymbol{W}(t) \right) \\ &= \boldsymbol{W}(t) - \alpha \boldsymbol{M}^T (\boldsymbol{M}\boldsymbol{W}(t) - \boldsymbol{I}) \boldsymbol{\tau}(t) \boldsymbol{\tau}(t)^T - \beta \boldsymbol{W}(t) \end{aligned} \quad \text{(A5)}$$

This equation can be averaged to give:

$$E\left[\boldsymbol{W}(t+1)\right] = \boldsymbol{W}(t) - \alpha \boldsymbol{M}^T (\boldsymbol{M}\boldsymbol{W}(t) - \boldsymbol{I})\boldsymbol{C} - \beta \boldsymbol{W}(t) \quad \text{(A6)}$$

where $\boldsymbol{C} = E[\boldsymbol{\tau}(t)\boldsymbol{\tau}(t)^T]$.

2.1. Uniform presentation of targets

If we assume that the targets are presented randomly and uniformly in space, $\boldsymbol{C}$ becomes $c\boldsymbol{I} \in \Re^{2\times 2}$ (c = 0.5, if the eight targets in Figure 1A are used) and Eq. (A6) becomes:

$$E\left[\boldsymbol{W}(t+1)\right] = ((1-\beta)\boldsymbol{I} - c\alpha \boldsymbol{M}^T \boldsymbol{M})\boldsymbol{W}(t) + c\alpha \boldsymbol{M}^T \quad \text{(A7)}$$

Using following substitutions:

$$\begin{cases} (1-\beta)\boldsymbol{I} - c\alpha \boldsymbol{M}^T \boldsymbol{M} = \boldsymbol{Q} \\ c\alpha \boldsymbol{M}^T = \boldsymbol{R} \end{cases} \quad \text{(A8)}$$

we can express Eq. (A7) as follows:

$$E\left[\boldsymbol{W}(t+1)\right] = \boldsymbol{Q}\boldsymbol{W}(t) + \boldsymbol{R} \quad \text{(A9)}$$

Thus, $\boldsymbol{W}(t)$ at the tth trial can be generally written as:

$$\boldsymbol{W}(t) = \boldsymbol{Q}^t \boldsymbol{W}(0) + (\boldsymbol{Q}^{t-1} + \boldsymbol{Q}^{t-2} + \cdots + \boldsymbol{Q} + \boldsymbol{I})\boldsymbol{R} \quad \text{(A10)}$$

Because $\boldsymbol{Q}^t$ can be expressed in the following form:

$$\boldsymbol{Q}^t = \boldsymbol{V} \begin{bmatrix} ((1-\beta) - c\alpha\lambda_1)^t & & & \cdots & 0 \\ & ((1-\beta) - c\alpha\lambda_2)^t & & & \vdots \\ & & (1-\beta)^t & & \\ \vdots & & & \ddots & \\ 0 & \cdots & & & (1-\beta)^t \end{bmatrix} \boldsymbol{V}^{-1} \quad \text{(A11)}$$

where λ_1 and λ_2 are the eigenvalues of the symmetric matrix $\boldsymbol{M}^T\boldsymbol{M}$, as the number of trials approaches infinity ($t \rightarrow \infty$),

$$\boldsymbol{Q}^t \rightarrow \boldsymbol{0} \tag{A12}$$

if the following conditions are satisfied:

$$\begin{cases} -1 < (1-\beta) - c\alpha\lambda_1 < 1 \\ -1 < (1-\beta) - c\alpha\lambda_2 < 1 \\ -1 < (1-\beta) < 1 \end{cases} \tag{A13}$$

Because α, λ_1, and λ_2 are positive values, the conditions in (A13) can be more simply written as follows:

$$\begin{cases} \beta < 2 - c\alpha\lambda_1 \\ \beta < 2 - c\alpha\lambda_2 \\ \beta > 0 \end{cases} \tag{A14}$$

Furthermore, under these conditions, as the number of trials approaches infinity ($t \rightarrow \infty$):

$$\boldsymbol{Q}^{t-1} + \boldsymbol{Q}^{t-2} + \cdots + \boldsymbol{Q} + \boldsymbol{I} \rightarrow (\boldsymbol{I} - \boldsymbol{Q})^{-1} \tag{A15}$$

From (A10), (A12), and (A15), as $t \rightarrow \infty$:

$$\begin{aligned} \boldsymbol{W}(t) &\rightarrow (\boldsymbol{I} - \boldsymbol{Q})^{-1}\boldsymbol{R} \\ &= (\boldsymbol{I} - \{(1-\beta)\boldsymbol{I} - c\alpha\boldsymbol{M}^T\boldsymbol{M}\})^{-1} c\alpha\boldsymbol{M}^T \\ &= (\frac{\beta}{c\alpha}\boldsymbol{I} + \boldsymbol{M}^T\boldsymbol{M})^{-1}\boldsymbol{M}^T \\ &= \boldsymbol{M}^T(\frac{\beta}{c\alpha}\boldsymbol{I} + \boldsymbol{M}\boldsymbol{M}^T)^{-1} \end{aligned} \tag{A16}$$

When $c\alpha \gg \beta$, $\boldsymbol{W}(t)$ converges to $\boldsymbol{M}^T(\boldsymbol{M}\boldsymbol{M}^T)^{-1}$, which is the Moore-Penrose pseudo-inverse of $\boldsymbol{M}$. Taken together, the conditions for convergence to the Moore-Penrose pseudo-inverse are:

$$\begin{cases} \beta < 2 - c\alpha\lambda_1 \\ \beta < 2 - c\alpha\lambda_2 \\ 0 < \beta << c\alpha \end{cases} \quad \text{(A17)}$$

The conditions in (A17) are satisfied, because α = 20, β = 1.0 × 10^{-4}, c = 0.5, λ_1 = 0.0036, and λ_2 = 0.00072.

Although we have proven the convergence of ***W*** for the 2-dimensional task, the proof also holds true for 3-dimensional or much higher-dimensional tasks. For a D-dimensional task, $\boldsymbol{W} \in \Re^{n\times D}$ converges to the Moore-Penrose pseudo-inverse of $\boldsymbol{M} \in \Re^{D\times n}$ under the following conditions:

$$\begin{cases} \beta < 2 - c\alpha\lambda_1 \\ \beta < 2 - c\alpha\lambda_2 \\ \quad \vdots \\ \beta < 2 - c\alpha\lambda_D \\ 0 < \beta << c\alpha \end{cases} \quad \text{(A18)}$$

2.2. Non-uniform presentation of targets

If the distribution of the targets is not uniform in space, ***C*** does not take the form $c\boldsymbol{I} \in \Re^{2\times 2}$. A transformation is conducted as follows:

$$\hat{\boldsymbol{\tau}}(t) = \boldsymbol{\sigma V \tau}(t) \quad \text{(A19)}$$

where $\boldsymbol{V} = (\boldsymbol{v}_1\boldsymbol{v}_2)$ is a matrix that consists of the eigenvectors of the matrix ***C***, and

$$\boldsymbol{\sigma} = \begin{pmatrix} 1/\sqrt{\lambda_1} & 0 \\ 0 & 1/\sqrt{\lambda_2} \end{pmatrix} \quad \text{(A20)}$$

where λ_1 and λ_2 are the eigenvalues. Then, $\hat{\boldsymbol{C}} = E\left[\hat{\boldsymbol{\tau}}(t)\hat{\boldsymbol{\tau}}(t)^T\right]$ becomes $\boldsymbol{I} \in \Re^{2\times 2}$, if there are at least two target vectors that are linearly independent.

In addition to the targets, the MDVs must be transformed in the same manner, $\hat{\boldsymbol{M}} = \boldsymbol{\sigma V M}$. When the synaptic weights from the new input vectors $\hat{\boldsymbol{\tau}}(t)$ to

the actuators are defined as $\hat{\boldsymbol{W}}$ and the output vectors as $\hat{\boldsymbol{T}}$, the transformed network has the same form as the original network. Therefore $\hat{\boldsymbol{W}}(t)$ converges to the pseudo-inverse of $\hat{\boldsymbol{M}}$:

$$\hat{\boldsymbol{W}}(t) \to \hat{\boldsymbol{M}}^T(\hat{\boldsymbol{M}}\hat{\boldsymbol{M}}^T)^{-1} \qquad \text{(A21)}$$

By substituting $\hat{\boldsymbol{M}} = \boldsymbol{\sigma V M}$ into Eq. (A21), we obtain:

$$\begin{aligned}\hat{\boldsymbol{W}}(t) &\to (\boldsymbol{\sigma VM})^T(\boldsymbol{\sigma VM}(\boldsymbol{\sigma VM})^T)^{-1} \\ &= \boldsymbol{M}^T(\boldsymbol{MM}^T)^{-1}(\boldsymbol{\sigma V})^{-1}\end{aligned} \qquad \text{(A22)}$$

Because the original $\boldsymbol{W}$ is expressed as:

$$\boldsymbol{W} = \hat{\boldsymbol{W}}\boldsymbol{\sigma V} \qquad \text{(A23)}$$

$\boldsymbol{W}(t)$ converges to the Moore-Penrose pseudo-inverse of $\boldsymbol{M}$ as follows:

$$\begin{aligned}\boldsymbol{W}(t) &\to \hat{\boldsymbol{W}}\boldsymbol{\sigma V} \\ &= \boldsymbol{M}^T(\boldsymbol{MM}^T)^{-1}(\boldsymbol{\sigma V})^{-1}\boldsymbol{\sigma V} \\ &= \boldsymbol{M}^T(\boldsymbol{MM}^T)^{-1}\end{aligned} \qquad \text{(A24)}$$

2.3. "Feedback-only" rule

Here, we mathematically assess where the synaptic weight matrix $\boldsymbol{W}(t)$ converges if the decay is not incorporated. As has been already shown, $\boldsymbol{W}(t)$ at the tth trial can be written as:

$$\boldsymbol{W}(t) = \boldsymbol{Q}^t\boldsymbol{W}(0) + (\boldsymbol{Q}^{t-1} + \boldsymbol{Q}^{t-2} + \cdots + \boldsymbol{Q} + \boldsymbol{I})\boldsymbol{R} \qquad \text{(A10)}$$

If the decay is not incorporated (i.e., $\beta = 0$), $\boldsymbol{Q}^t$ is expressed as follows:

$$\boldsymbol{Q}^t = \boldsymbol{V}\begin{bmatrix}(1-c\alpha\lambda_1)^t & & & \cdots & 0 \\ & (1-c\alpha\lambda_2)^t & & & \vdots \\ & & 1^t & & \\ \vdots & & & \ddots & \\ 0 & \cdots & & & 1^t\end{bmatrix}\boldsymbol{V}^{-1} \qquad \text{(A25)}$$

where λ_1 and λ_2 are the eigenvalues of the symmetric matrix $\boldsymbol{M}^T\boldsymbol{M}$ and the matrix $\boldsymbol{V}$ (= ($\mathbf{v}_1$ $\mathbf{v}_2$ … $\mathbf{v}_n$)) consists of the eigenvectors of the symmetric matrix $\boldsymbol{M}^T\boldsymbol{M}$. When (A14) is satisfied, as the number of trials approaches infinity ($t \to \infty$):

$$\boldsymbol{Q}^t \to \boldsymbol{V}\begin{bmatrix} 0 & & & \cdots & 0 \\ & 0 & & & \vdots \\ & & 1 & & \\ \vdots & & & \ddots & \\ 0 & \cdots & & & 1 \end{bmatrix}\boldsymbol{V}^{-1} = \boldsymbol{A}(\neq \boldsymbol{0}) \qquad \text{(A26)}$$

Thus, $|\boldsymbol{AW}(0)| \leq |\boldsymbol{W}(0)|$, which means that $\boldsymbol{A}$ never increases |$\boldsymbol{W}$(0)|. As for the second term of Eq.(A10), as the number of trials approaches infinity ($t \to \infty$):

$$\begin{aligned}(\boldsymbol{Q}^{t-1} + \boldsymbol{Q}^{t-2} + \cdots + \boldsymbol{Q} + \boldsymbol{I})\boldsymbol{R} &\to (\boldsymbol{I} - \boldsymbol{Q})^{-1}\boldsymbol{R} \\ &= \boldsymbol{M}^T(\frac{\beta}{c\alpha}\boldsymbol{I} + \boldsymbol{MM}^T)^{-1}\end{aligned} \qquad \text{(A27)}$$

When β = 0, it converges to $\boldsymbol{M}^T(\boldsymbol{MM}^T)^{-1}$. Taken together, the synaptic weight matrix converges as:

$$\boldsymbol{W}(t) \to \boldsymbol{AW}(0) + \boldsymbol{M}^T(\boldsymbol{MM}^T)^{-1} \;\; (t \to \infty) \qquad \text{(A28)}$$

Finally, $\boldsymbol{MW}(t)$ converges on:

$$\begin{aligned}\boldsymbol{MW}(t) &\to \boldsymbol{M}[\boldsymbol{AW}(0) + \boldsymbol{M}^T(\boldsymbol{MM}^T)^{-1}] \;\; (t \to \infty) \\ &= \boldsymbol{MAW}(0) + \boldsymbol{I}\end{aligned} \qquad \text{(A29)}$$

Because $\boldsymbol{M}$ can be decomposed using singular value decomposition as follows:

$$\boldsymbol{M} = \boldsymbol{U}\begin{bmatrix} \sqrt{\lambda_1} & 0 & 0 & \cdots & 0 \\ 0 & \sqrt{\lambda_2} & 0 & \cdots & 0 \end{bmatrix}\boldsymbol{V}^T \qquad \text{(A30)}$$

where the matrix $\boldsymbol{U}$ (= ($\mathbf{u}_1$ $\mathbf{u}_2$)) includes the eigenvectors of the symmetric matrix $\boldsymbol{MM}^T$. Therefore, $\boldsymbol{MAW}(0)$ is calculated as:

$$\boldsymbol{MAW}(0) = \left(\boldsymbol{U} \begin{bmatrix} \sqrt{\lambda_1} & 0 & 0 & \cdots & 0 \\ 0 & \sqrt{\lambda_2} & 0 & \cdots & 0 \end{bmatrix} \boldsymbol{V}^T \right) \left(\boldsymbol{V} \begin{bmatrix} 0 & & & \cdots & 0 \\ & 0 & & & \vdots \\ & & 1 & & \\ \vdots & & & \ddots & \\ 0 & \cdots & & & 1 \end{bmatrix} \boldsymbol{V}^{-1} \right) \boldsymbol{W}(0)$$

Because $\boldsymbol{V}$ is a matrix containing the eigenvectors of the symmetric matrix $\boldsymbol{M}^T\boldsymbol{M}$, $\boldsymbol{V}$ is an orthogonal matrix satisfying $\boldsymbol{V}^T\boldsymbol{V} = \boldsymbol{I}$. Therefore, it is further calculated as:

$$\boldsymbol{MAW}(0) = \boldsymbol{U} \begin{bmatrix} \sqrt{\lambda_1} & 0 & 0 & \cdots & 0 \\ 0 & \sqrt{\lambda_2} & 0 & \cdots & 0 \end{bmatrix} \begin{bmatrix} 0 & & & \cdots & 0 \\ & 0 & & & \vdots \\ & & 1 & & \\ \vdots & & & \ddots & \\ 0 & \cdots & & & 1 \end{bmatrix} \boldsymbol{V}^{-1}\boldsymbol{W}(0)$$

$$= \boldsymbol{U} \begin{bmatrix} 0 & 0 & 0 & \cdots & 0 \\ 0 & 0 & 0 & \cdots & 0 \end{bmatrix} \boldsymbol{V}^{-1}\boldsymbol{W}(0) = \boldsymbol{0} \qquad \text{(A31)}$$

Note that $\boldsymbol{AW}(0)$ itself is not $\boldsymbol{0}$ and depends on the initial synaptic weight $\boldsymbol{W}(0)$, but it always satisfies $\boldsymbol{MAW}(0) = \boldsymbol{0}$, irrespective of the initial synaptic weight matrix. From (A29) and (A31), as $t \to \infty$:

$$\boldsymbol{MW}(t) \to \boldsymbol{I} \qquad \text{(A32)}$$

Thus, the converged weight matrix produces zero error for the arbitrary target $\boldsymbol{\tau}$ because it always satisfies $\boldsymbol{T} = \boldsymbol{MW\tau} = \boldsymbol{\tau}$.

In summary, by the “feedback-only” rule, the synaptic weight matrix $\boldsymbol{W}(t)$ converges with a different matrix depending on the initial synaptic weight matrix (i.e., $\boldsymbol{W}(t) \to \boldsymbol{AW}(0) + \boldsymbol{M}^T(\boldsymbol{MM}^T)^{-1}$), while the converged weight matrices always satisfy $\boldsymbol{MW} = \boldsymbol{I}$ and hence produce zero error for the arbitrary target.
